# WASP and SCAR are evolutionarily conserved in actin-filled pseudopod-based motility

**DOI:** 10.1083/jcb.201701074

**Published:** 2017-06-05

**Authors:** Lillian K. Fritz-Laylin, Samuel J. Lord, R. Dyche Mullins

**Affiliations:** Department of Cellular and Molecular Pharmacology, Howard Hughes Medical Institute, University of California, San Francisco, San Francisco, CA 94143

## Abstract

Diverse eukaryotic cells crawl through complex environments using distinct modes of migration. To understand the underlying mechanisms and their evolutionary relationships, we must define each mode and identify its phenotypic and molecular markers. In this study, we focus on a widely dispersed migration mode characterized by dynamic actin-filled pseudopods that we call “α-motility.” Mining genomic data reveals a clear trend: only organisms with both WASP and SCAR/WAVE—activators of branched actin assembly—make actin-filled pseudopods. Although SCAR has been shown to drive pseudopod formation, WASP’s role in this process is controversial. We hypothesize that these genes collectively represent a genetic signature of α-motility because both are used for pseudopod formation. WASP depletion from human neutrophils confirms that both proteins are involved in explosive actin polymerization, pseudopod formation, and cell migration. WASP and WAVE also colocalize to dynamic signaling structures. Moreover, retention of WASP together with SCAR correctly predicts α-motility in disease-causing chytrid fungi, which we show crawl at >30 µm/min with actin-filled pseudopods. By focusing on one migration mode in many eukaryotes, we identify a genetic marker of pseudopod formation, the morphological feature of α-motility, providing evidence for a widely distributed mode of cell crawling with a single evolutionary origin.

## Abstract (precis)

Eukaryotic cells use diverse cellular mechanisms to crawl through complex environments. Fritz-Laylin et al. define α-motility as a mode of migration associated with dynamic, actin-filled pseudopods and show that WASP and SCAR constitute an evolutionarily conserved genetic signature of α-motility.

## Introduction

Eukaryotic cells move using several distinct modes of locomotion, including crawling and flagella-driven swimming. The stereotyped architecture of flagella and the conservation of their protein components make the evolutionary conservation of cell swimming clear. In contrast, “crawling motility” is a collection of distinct processes whose evolutionary relationships are not well understood (Rodriguez et al., 2005; Lämmermann and Sixt, 2009; Paluch and Raz, 2013). Some crawling cells require dedicated adhesion molecules to make specific, high-affinity contacts with their surroundings, whereas other cells rely on weaker, nonspecific interactions. Crawling cells also use different mechanisms to advance their leading edge, either assembling polymerized actin networks to push the plasma membrane forward or detaching the membrane from the underlying cytoskeleton to form a rapidly expanding bleb. Furthermore, some cell types have been shown to use contractile forces to generate forward movement (Lämmermann et al., 2008; Bergert et al., 2012; Liu et al., 2015). Different cells can also use different sets of molecules to drive similar modes of crawling. In an extreme example, nematode sperm have evolved a method of crawling in which polymer assembly advances the leading-edge membrane, but in these cells, the force-generating polymer networks are composed of major sperm protein rather than actin (Rodriguez et al., 2005). Given this variety of crawling behaviors, it is clear that one cannot simply assume that the underlying molecular mechanisms are the same.

The best-understood mode of crawling is the slow (1–10 µm/h) creeping of adherent animal cells, including fibroblasts and epithelial cells (Petrie and Yamada, 2015). These cells move by extending across a surface a sheet-like protrusion called a lamellipodium while also gripping substrate molecules using integrins, which are often clustered into large focal adhesions. Although clinically and physiologically important, this form of adhesion-based crawling is unique to the animal lineage and is largely restricted to molecular highways formed by the extracellular matrix.

In contrast, many motile cells—including free-living amoebae and human immune cells—make 3D actin-filled pseudopods and navigate complex environments at speeds exceeding 20 µm/min (100–1,000× faster than fibroblasts) without forming specific molecular adhesions (Buenemann et al., 2010; Butler et al., 2010). Although this mode of fast cell crawling has been called “ameboid motility,” this term is also used to describe a range of behaviors, including cell motility that relies on membrane blebs rather than actin-filled pseudopods (Lämmermann and Sixt, 2009).

To narrow our focus, we use the term “α-motility” specifically to describe cell crawling that is characterized by: (i) highly dynamic 3D pseudopods at the leading edge that are filled with branched actin networks assembled by the Arp2/3 complex; (ii) fast migration typically on the order of tens of µm/min; and (iii) the absence of specific, high-affinity adhesions to the extracellular environment. This independence from specific molecular adhesions separates α-motility from the adhesion-based motility of fibroblasts and epithelial cells. Furthermore, the use of pseudopods discriminates it from the fast bleb-based motility adopted by fibroblasts in environments that preclude adhesion formation (Liu et al., 2015; Ruprecht et al., 2015). Some organisms using α-motility may also use additional methods of generating forward movement, such as contractility, retrograde flow, and/or blebbing (Yoshida and Soldati, 2006; Lämmermann et al., 2008; Bergert et al., 2012), but in this study, we focus on a single phenotype readily observable in diverse species, including nonmodel organisms.

Organisms with cells capable of α-motility appear throughout the eukaryotic tree, and we hypothesize that this form of locomotion reflects a single, discrete process that arose early in eukaryotic evolution and has been conserved. If this hypothesis is correct, then elements of this ancient process—specific molecules and mechanisms—may be conserved and still associated with cell crawling in distantly related organisms that use α-motility. Such molecular remnants would help to unravel the evolutionary history of cell locomotion and might enable us to predict the existence of specific modes of motility in poorly characterized species. Identifying genes associated with a process such as α-motility is not trivial because the core machinery driving pseudopod formation (e.g., actin and the Arp2/3 complex) is shared with other cellular processes, including some types of endocytosis (Winter et al., 1997). The participation of these proteins in multiple essential processes likely explains their ubiquity in the eukaryotic family tree. Actin, for example, is found in the genomes of all eukaryotes and, based on phylogenetic analysis, is widely accepted to have been present in the eukaryotic ancestor (Goodson and Hawse, 2002). The Arp2/3 complex is also highly conserved (Beltzner and Pollard, 2004). It is present in the genomes of all sequenced eukaryotes except the parasitic protist *Giardia intestinalis*, whose lineage either split off before the evolution of the Arp2/3 complex or lost it, depending on the placement of the root of the eukaryotic tree (Paredez et al., 2011). The ubiquity and multifunctionality of actin and the Arp2/3 complex make them difficult to use as markers for tracing the evolutionary history of α-motility.

We therefore turned our attention to upstream regulators of actin assembly. There are several nucleation-promoting factors that stimulate branched actin network assembly by the Arp2/3 complex in response to various upstream cellular signals (Rottner et al., 2010). Some of these Arp2/3 activators are restricted to specific eukaryotic lineages, particularly multicellular animals that evolved JMY and WHAMM families of Arp2/3 activators, whereas other activators are more widely distributed (Veltman and Insall, 2010; Kollmar et al., 2012). For example, WASP and SCAR (also known as WAVE) are widely conserved Arp2/3 activators that respond to different signaling cascades (Rohatgi et al., 1999; Moreau et al., 2000; Koronakis et al., 2011) and promote different levels of Arp2/3 activity (Zalevsky et al., 2001). Multiple published phylogenetic analyses of WASP and SCAR gene families suggest that both genes are ancient and likely to have been present in the eukaryotic ancestor (Veltman and Insall, 2010; Kollmar et al., 2012). However, neither family is found in all eukaryotic lineages, making them appealing candidates for genetic markers of α-motility.

SCAR is generally accepted to play a major role in the formation of protrusions used for cell motility (Miki et al., 1998; Steffen et al., 2004; Weiner et al., 2006; Veltman et al., 2012). In contrast, the involvement of WASP genes (particularly the two mammalian homologues WASP and N-WASP) in cell crawling is less clear. N-WASP is ubiquitously expressed in mammals and is dispensable for lamellipodia or filopodia formation by adherent fibroblasts (Lommel et al., 2001; Snapper et al., 2001; Sarmiento et al., 2008), which has led many researchers to discount a role for any WASP protein in protrusions or motility (Small and Rottner, 2010; Veltman and Insall, 2010). Mammalian WASP, however, is expressed only in blood cells, where it has been shown to be involved in migration and pseudopod formation (Badolato et al., 1998; Burns et al., 2001; Jones et al., 2002, 2013; Shi et al., 2009; Ishihara et al., 2012). Further evidence for a role of WASP in cell migration comes from the handful of papers studying WASP in nonmammalian cells (Veltman et al., 2012; Zhu et al., 2016; and see Tables S1 and S2 for an annotated bibliography of the 29 papers on WASP and N-WASP relating to cell migration summarized here). The use of WASP by highly motile cells but not by adherent fibroblasts may therefore reflect unique requirements for α-motility.

To understand the regulation of the actin cytoskeleton during pseudopod formation, we exploit the diversity of organisms that use α-motility. By comparing the genomes of many eukaryotes, we find that organisms with genes encoding both WASP and SCAR make pseudopods, and organisms that do not build pseudopods have lost either or both Arp2/3 activators. We validate this molecular signature using a negative test (depleting the protein disrupts pseudopod formation in well-studied cells) as well as a positive test (a new prediction of α-motility in a little-studied organism). Differentiating α-motility from slow/adhesive cell migration helps clarify the confusion over WASP’s importance in cell motility and shifts the major question from whether WASP or SCAR is required for motility in a given single cell type to how WASP and SCAR work together to construct and maintain pseudopods in many species. The retention of WASP and SCAR by organisms that form pseudopods represents the first molecular support, to our knowledge, for a single origin of this widespread form of cell motility in an ancestor of extant eukaryotes (see Fig. 8 for summary).

## Results

### Evolutionary retention of both WASP and SCAR correlates with pseudopod formation

To trace the evolutionary history of α-motility, we first determined which sequenced eukaryotic organisms might use α-motility. The obvious structural features associated with α-motility are dynamic, actin-filled pseudopods. In addition to α-motility, some organisms use these structures for feeding. Pseudopods used for feeding and α-motility share so many structural and signaling components that unless specific receptors and/or prey are known to be involved, they are largely indistinguishable (Heinrich and Lee, 2011). Therefore, we combed the literature for references to organisms with cells that form pseudopods for feeding and/or α-motility. Eukaryotic phyla fall into at least six large clades, and species with sequenced genomes and that form pseudopods can be found in most (see Fig. 8 and Table 1).

**Table 1.** SCAR and WASP orthologues for indicated species (Kollmar et al., 2012) for protein sequences (except for orthologues in *Sarracenia rosea*, which we identified via BLAST, NCBI identifiers indicated). See also Fig. 8.

**Species**	**Group**	SCAR	**WASP**	**Pseudopod reference**
*H. sapiens*	Animals (opisthokont)	WAVE1, WAVE2, WAVE3	WASP, N-WASP	Ramsey, 1972
*M. brevicollis* (orthologues in *S. rosea*)	Choanoflagellate (opisthokont)	MbWAVE (*S. rosea*: XP_004994362)	MbWASP (*S. rosea:* XP_004997564)	Pseudopod-based feeding has been observed in all choanoflagellate families (Pettitt et al., 2002); for detailed analysis of pseudopods of *S. rosea* see Dayel and King, 2014
*C. owczarzaki*	Opisthokont	CoWAVE	CoWASP	Hertel et al., 2002
*A. nidulans* (Representative dikaryon)	Fungi (opisthokont)	n.f.	EnWASP	n.f.
*B. dendrobatidis*	Fungi (opisthokont)	Bad_bWAVE	Bad_bWASP	this work
*A. macrogynus*	Fungi (opisthokont)	AlmWAVE	AlmWASP_Aα, AlmWASP_Aβ, AlmWASP_Bα, AlmWASPB_β	n.f.
*D. discoideum*	Amoebozoan	SCAR1	DdWASP	Raper, 1935
*E. histolytica*	Amoebozoan	n.f.	n.f.	n.f.
*A. castellanii*	Amoebozoan	AcWAVE	AcWASP_A, AcWASP_B, AcWASP_C	Bowers and Korn, 1968
*T. trahens++*	Apusozoa	TctWAVE	TctWASP	Cavalier-Smith and Chao, 2010
*A. thaliana*	Plant	SCAR1, SCAR2, SCAR3, SCAR4, SCAR-like domain-containing protein	n.f.	n.f.
*P. ramorum*	Stramenopile (SAR)	n.f.	n.f.	n.f.
*B. natans*	Rhizarian (SAR)	n.f.	BinWASP_A, BinWASP_B	n.f.
*P. falciparum*	Alveolate (SAR)	n.f.	n.f.	n.f.
*T. thermophila*	Alveolate (SAR)	n.f.	TtWASP_A, TtWASP_B	n.f.
*E. huxleyi*	Haptophyte	n.f.	*Emh*Wasp	n.f.
*N. gruberi*	Heterolobosean	NgWAVE_A, NgWAVE_B	NgWASP_A, NgWASP_B, NgWASP_C, NgWASP_D	Fulton, 1970
*G. lamblia*	Diplomonad	n.f.	n.f.	n.f.
*T. vaginalis*	Parabasalid	Tv_aWAVE_A, Tv_aWAVE_B, Tv_aWAVE_C, Tv_aWAVE_D, Tv_aWAVE_E, Tv_aWAVE_F, Tv_aWAVE_G, Tv_aWAVE_H, Tv_aWAVE_I, Tv_aWAVE_J, Tv_aWAVE_K	Tv_aWasp_A, Tv_aWasp_B	Kusdian et al., 2013

Example references for observations of pseudopods are given. Cases where genes or references to cells with active pseudopods were not found are indicated by “n.f.” See also Fig. 8.

On to this map of the phylogenetic distribution of pseudopods we overlaid the conservation of WASP and SCAR/WAVE genes, using a recently published manually curated database of nucleation-promoting factors from genomes spanning eukaryotic diversity (Kollmar et al., 2012). Multiple analyses have concluded that both WASP and SCAR were present in the last common ancestor of eukaryotes (Veltman and Insall, 2010; Kollmar et al., 2012) and therefore argue that a lack of either gene reflects loss during evolution.

To understand whether these gene loss events reveal a significant pattern, we compared the conservation of individual nucleation-promoting factors across large evolutionary distances with the ability to assemble pseudopods. We identified a correlation between the conservation of WASP and SCAR and pseudopod formation (see Fig. 8 and Table 1).

For example, no plant cells build pseudopods, and no sequenced plant genomes contain a WASP orthologue. Similarly, multicellular fungi—the dikarya—lack SCAR and are also not known to build pseudopods. Conversely, almost all sequenced genomes of *Amoebozoan* species (including dictyostelids) encode orthologues of WASP and SCAR, and almost all move with the help of dynamic, actin-rich pseudopods. A potential counterexample is the amoeba *Entamoeba histolytica*, which lacks both WASP and SCAR but forms Arp2/3-dependent phagocytic “food cups” to engulf bacteria (Babuta et al., 2015). The absence of both genes indicates that *Entamoeba* must use another Arp2/3 activation system for protrusions, an idea supported by its use of blebs and not pseudopods for motility (Maugis et al., 2010). But the most glaring exception to the correlation was a pair of little-studied species of chytrid fungi that retain both nucleation-promoting factors but are not known to build pseudopods: *Allomyces macrogynus* and *Batrachochytrium dendrobatidis* (Bd).

We took a two-pronged approach to testing our hypothesis that retention of WASP together with SCAR serves as a molecular signature of pseudopod formation. First, we took the more traditional approach and confirmed that both genes are involved in pseudopod formation in mammalian cells. We followed this with an evolution-based approach by verifying the ability of this molecular signature to predict the capacity for pseudopod formation in chytrid fungi.

### WASP and SCAR localize to the same dynamic arcs within pseudopods of human neutrophils

Our evolutionary evidence indicates that WASP and SCAR may both be required to build pseudopods. To test this hypothesis directly, we turned to human cell lines capable of forming pseudopods. HL-60 cells are derived from an acute myeloid leukemia (Collins et al., 1977) and retain many features of hematopoietic cells, including expression of hematopoietic WASP and the capacity to differentiate into fast-migrating neutrophils with dynamic pseudopods (Collins et al., 1978).

To follow the dynamics of WASP localization in live cells, we created an HL-60 line stably expressing full-length WASP fused at the N terminus to the red fluorescent protein TagRFP-T. By confocal fluorescence microscopy, TagRFP-WASP concentrated in two distinct locations within migrating HL-60 cells: punctate foci distributed throughout the cell and a broad zone near the leading edge (Fig. S1 A).

A previous study has shown that the SCAR regulatory complex localizes to fast-moving anterograde “waves” that break against the leading edge of actively migrating HL-60 cells (Weiner et al., 2007). This localization pattern is most easily observed using total internal reflection fluorescence (TIRF) microscopy, which illuminates a ∼100-nm thick region of the cell near the ventral surface (Axelrod, 1981). Using TIRF microscopy on rapidly migrating HL-60 cells, we observed that TagRFP-WASP concentrates near the leading edge in linear arcs that move in an anterograde direction similar to previously observed patterns of the SCAR regulatory complex (Weiner et al., 2007).

**Figure 1.**
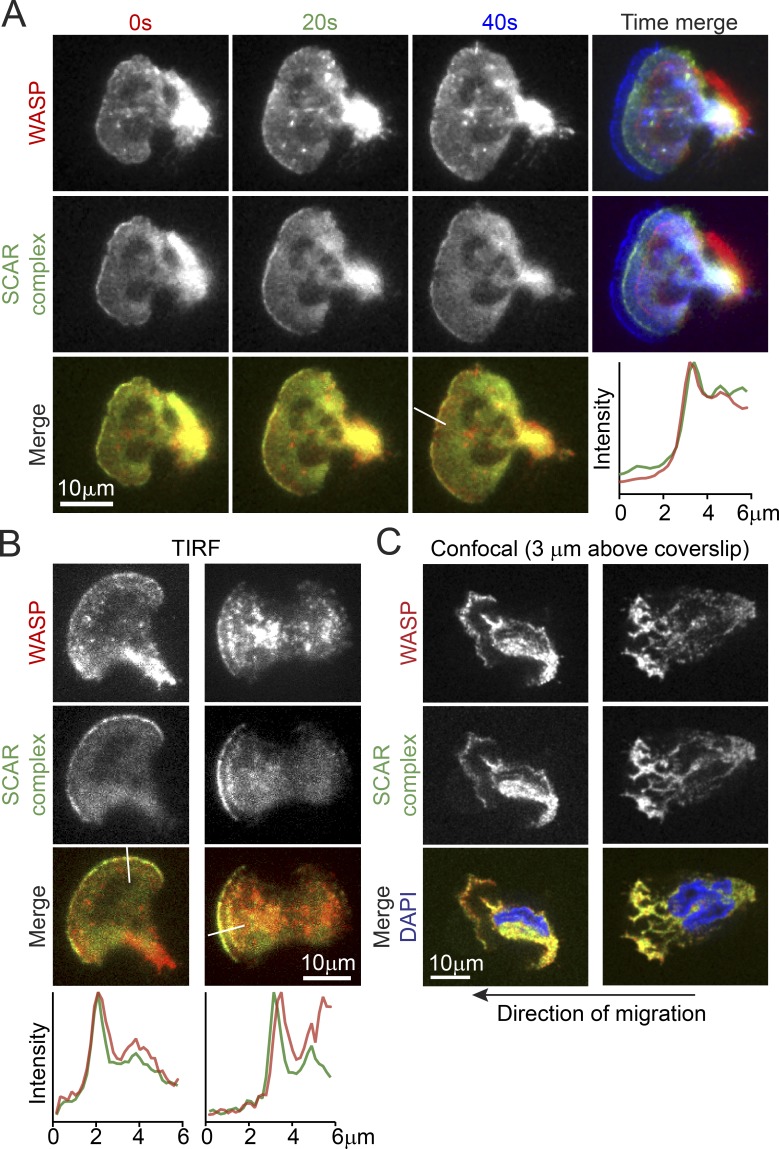
**WASP colocalizes with the SCAR complex at the leading edge of neutrophils.** Microscopy of HL-60 cells expressing TagRFP-WASP and Hem-1–YFP, a component of the SCAR regulatory complex. (A) TIRF images of live HL-60 cells. Top: WASP localization in three sequential time points, overlaid on the far right (0 s in red, 20 s in green, and 40 s in blue). Middle: same sequence of images but for Hem-1. Bottom: overlay of WASP and Hem-1 at each time point. The plot shows line scans of normalized fluorescence intensity of WASP (red) and Hem-1 (green). The location for generating the line scans is shown in the adjacent image (white line). (B) Two additional examples of live HL-60 cells in TIRF and corresponding line scans (indicated by white lines in the images). (C) Spinning-disk confocal images of two fixed HL-60 cells showing an axial slice through the middle of thick pseudopods. The slices shown were taken 3 µm above the coverslip. See also Fig. S1 (for additional images and kymographs) and Video 1. For all cells, the direction of migration is to the left.

To see whether WASP and SCAR travel together in the same waves, we introduced TagRFP-WASP into cells expressing YFP-Hem1, a core component of the SCAR regulatory complex (Weiner et al., 2007). TIRF microscopy of these cells revealed that WASP and the SCAR regulatory complex move together in the same dynamic, linear arcs (Fig. 1, A and B; Fig. S1 B; and Video 1). Interestingly, however, the localization patterns of the two are not identical, an observation confirmed by quantifying WASP and SCAR localization across the leading edge (Fig. S1 C). Spinning-disk confocal microscopy indicated that WASP and SCAR colocalize throughout the growing pseudopods, not only at the ventral surface (Fig. 1 C). Within the resolution limits of our imaging, the localization patterns moved together, with neither protein consistently leading the other (Fig. S1 B and Video 1). This dynamic localization pattern suggests that both WASP and SCAR activate the Arp2/3 complex in leading-edge pseudopods, promoting assembly of the branched actin networks required for membrane protrusion.

### WASP participates in pseudopod assembly in neutrophils

**Figure 2.**
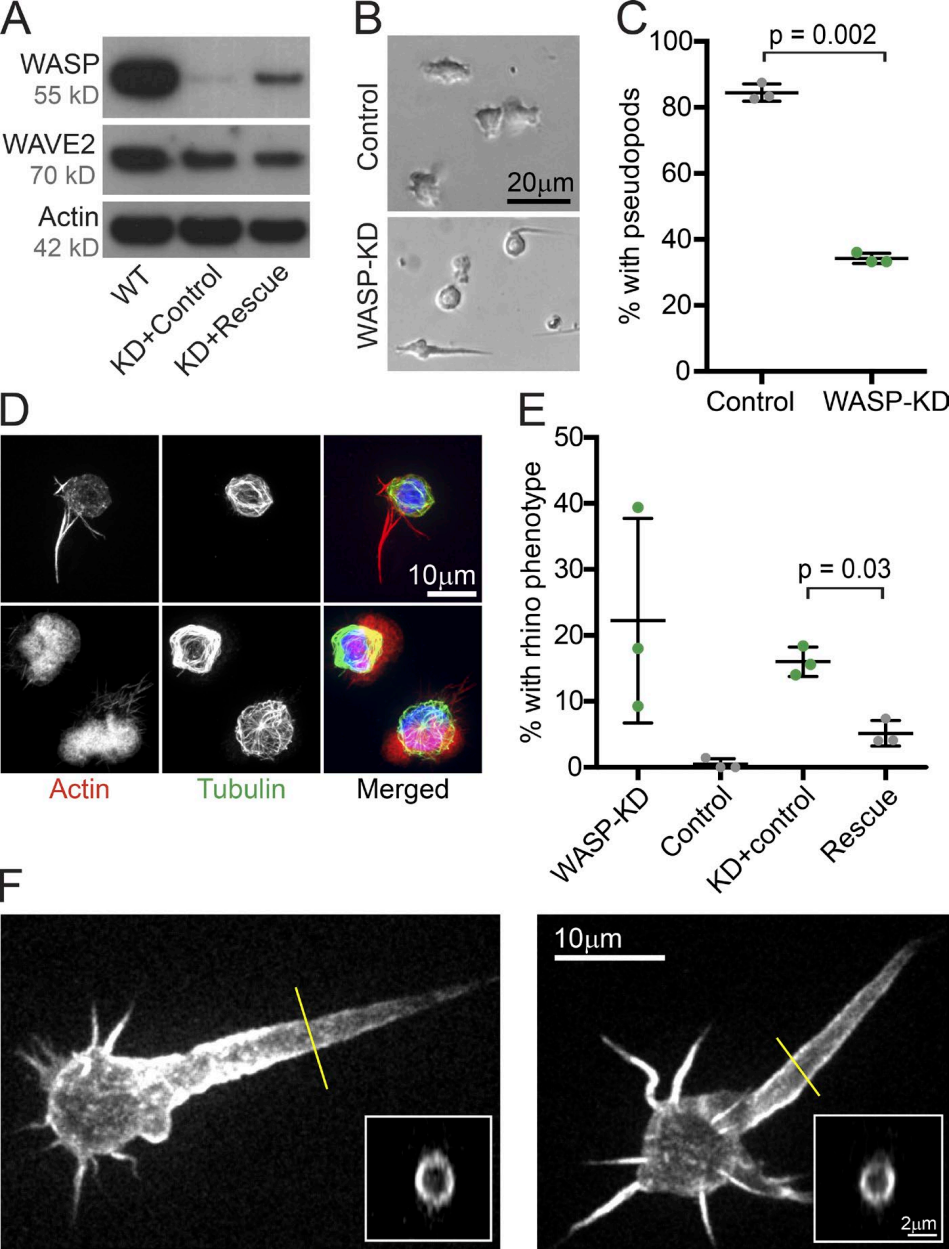
**WASP is crucial for pseudopod formation of neutrophils.** (A) Western blots showing WASP and WAVE2 expression in control HL-60 cells, cells expressing shRNA to WASP, and exogenous WASP with three silent mutations in the region corresponding to the shRNA (KD + rescue) or cells expressing anti-WASP shRNA and empty vector rescue control (KD + control). Approximately equal amounts of total protein were loaded in each lane, which was confirmed by using actin as a loading control. (B) Brightfield images of control and WASP-KD cells. (C) KD of WASP using shRNA reduces the percentage of cells that have pseudopods. (D) Immunofluorescence of control and WASP-KD HL-60 cells showing microtubules (green, antibody stained), actin filaments (red, phalloidin stained), and DNA (blue, DAPI). Note the signature rhino phenotype in the WASP-KD cell. See also Fig. S2. (E) Rescue of rhino protrusion phenotype by expression of shRNA-insensitive WASP as described in A. Note: no wild-type (WT) cells were observed to exhibit the rhino phenotype. (C and E) Means and SD (bars) of three biological replicates (dots) with >130 cells total; p-values were obtained with two-tailed paired *t* tests. (F) Maximum projections of spinning-disk confocal stacks of living HL-60 cells with fluorescent probes specific for polymerized actin (mCherry fused to the calponin homology domain of Utrophin; Utr261). Insets are cross sections through the rhino horns at positions indicated by yellow lines, confirming that they are hollow with a shell of actin. See also Fig. S2 for a time lapse of the right-hand cell showing the dynamics of the rhino horn protrusion.

To investigate whether WASP is involved in pseudopod assembly by HL-60 cells, we generated anti-WASP shRNAs, expression of which resulted in a >90% reduction of WASP protein but no obvious change in WAVE2 (Fig. 2 A). We next examined whether WASP-depleted (WASP-knockdown; KD; WASP-KD) cells can form pseudopods. In a gradient of chemoattractant (the peptide fMet-Leu-Phe [fMLP]), wild-type HL-60 cells became strongly polarized, with broad, actin-rich pseudopods used to rapidly move toward the source of chemoattractant (Fig. 2 B and Video 2). Compared with control, 50% fewer WASP-KD cells formed pseudopods (Fig. 2, B and C). Despite numerous attempts, we never succeeded in developing WASP-KD cell lines in which this phenotype was 100% penetrant. Although it is possible that this was caused by residual WASP protein, this seems an insufficient explanation because the WASP-KD cells still capable of forming pseudopods were also aberrant (see the next section). Moreover, cells from WASP knockout mice also showed only a partial defect in gross cell motility in vivo (Snapper et al., 2005).

In addition to the defect in pseudopod formation, ∼20% of WASP-KD cells formed large protrusions that tapered to a point, reminiscent of a rhinoceros horn (Fig. 2, B, D, and F; Fig. S2; and Video 2; WASP-KD cells 11, 32, 33, 39, and 42, for example). To verify its specificity, we rescued this “rhino” phenotype by expressing a functional WASP containing three silent mutations in the sequence targeted by the shRNA (Fig. 2 E). Additionally, a second shRNA that targets a separate region of the WASP gene resulted in a significantly smaller effect on both WASP expression and the number of cells with the rhino phenotype (not depicted). Immunofluorescence combined with phalloidin staining of polymerized actin revealed that the aberrant rhino protrusions contained actin filaments but lacked microtubules (Fig. 2 D). The expression of a probe specific for polymerized actin (mCherry fused to the calponin homology domain of Utrophin; Utr261; Burkel et al., 2007) revealed a highly dynamic and surprisingly hollow actin filament network inside the protrusions (Figs. 2 F and S2). This distribution, enriched near the membrane but depleted from the core of the protrusion, is more reminiscent of cortical actin networks than of filopodia, which are packed tightly with actin bundles (Tilney et al., 1973).

Because some WASP family proteins contribute to endocytosis (Naqvi et al., 1998; Merrifield et al., 2004; Benesch et al., 2005), we investigated whether the defects in WASP-KD cells are caused by reduction of endocytosis. In undifferentiated HL-60s, we observed no difference in transferrin receptor endocytosis and recycling between WASP-KD and control cells (Fig. S3 E). After differentiation into cells capable of making pseudopods, WASP-KD HL-60s actually showed increased surface receptor densities (Fig. S3 D), receptor internalization (Fig. S3 B), and receptor recycling (Fig. S3 C) compared with control cells. Therefore, we cannot attribute the WASP-KD phenotypes simply to a curtailment of endocytosis activity.

### WASP-depleted neutrophils polymerize less actin in response to chemoattractant

**Figure 3.**
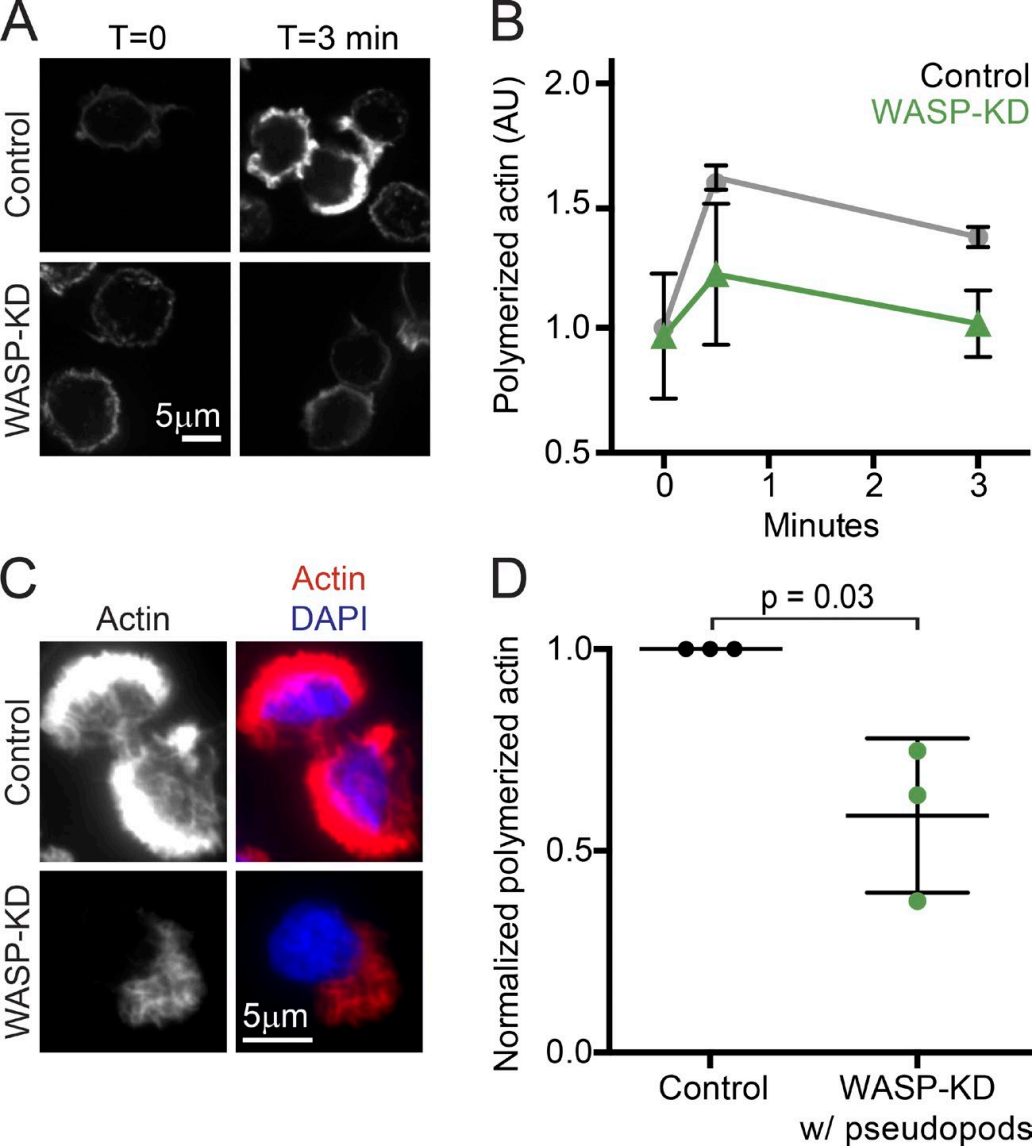
**WASP is crucial for explosive actin polymerization during pseudopod formation in neutrophils.** (A) Spinning-disk confocal images of polymerized actin of control and WASP-KD HL-60 cells after stimulation for the indicated time with chemoattractant (20 nM fMLP) stained with fluorescent phalloidin. (B) FACS quantification of actin polymerization in control (gray circles) and WASP-KD HL-60 cells (green triangles) after stimulation for the indicated time with chemoattractant stained with fluorescent phalloidin. 10,000 cells were counted for each sample, and means were normalized to time 0 for control cells within each experiment to control for detector variability. AU, arbitrary unit. (C) Example spinning-disk confocal images of pseudopod-forming WASP-KD and control cells with polymerized actin stained with phalloidin (red) and DNA stained with DAPI (blue). (D) Quantification of phalloidin staining shown in C. Only cells with pseudopods were analyzed. Mean pixel values for z projections of image stacks was measured for each cell, and then the mean background pixel value was subtracted. Means and SD (bars) from three biological replicates (dots) are shown, with >150 cells total; the p-value was obtained from a one-tailed paired *t* test.

Addition of chemoattractant to nonpolarized (quiescent) HL-60 cells induced a burst of actin polymerization that drove polarization and pseudopod formation, nearly doubling the cell’s polymerized actin content within 30 s of stimulation. This response is already known to depend on the activity of the SCAR regulatory complex (Weiner et al., 2006). To determine what role WASP might play in this explosive actin assembly, we synchronized pseudopod formation by stimulating populations of quiescent HL-60s with fMLP and then fixed and stained the cells with phalloidin at different time points and analyzed total polymerized actin content in each cell by confocal microscopy and FACS (Fig. 3, A and B). In the absence of chemoattractant, the amount of polymerized actin in quiescent WASP-KD cells was roughly equal to that in control cells. However, as reported for SCAR-depleted cells (Weiner et al., 2006), WASP-KD cells had greatly reduced actin polymerization at both short (30 s) and long times (3 min) after stimulation. This reduced actin polymerization indicates that WASP, like SCAR, is central to the explosive actin polymerization required for cell polarization and subsequent pseudopod formation.

To understand more about the pseudopods formed by some WASP-KD cells, we analyzed confocal images of hundreds of individual phalloidin-stained cells. Quantification of the actin content in the subset of WASP-KD cells that make pseudopods compared with control cells revealed that even WASP-KD cells that appeared to make “normal” pseudopods contained about half the quantity of polymerized actin (Fig. 3, C and D).

### WASP depletion impairs neutrophil motility

**Figure 4.**
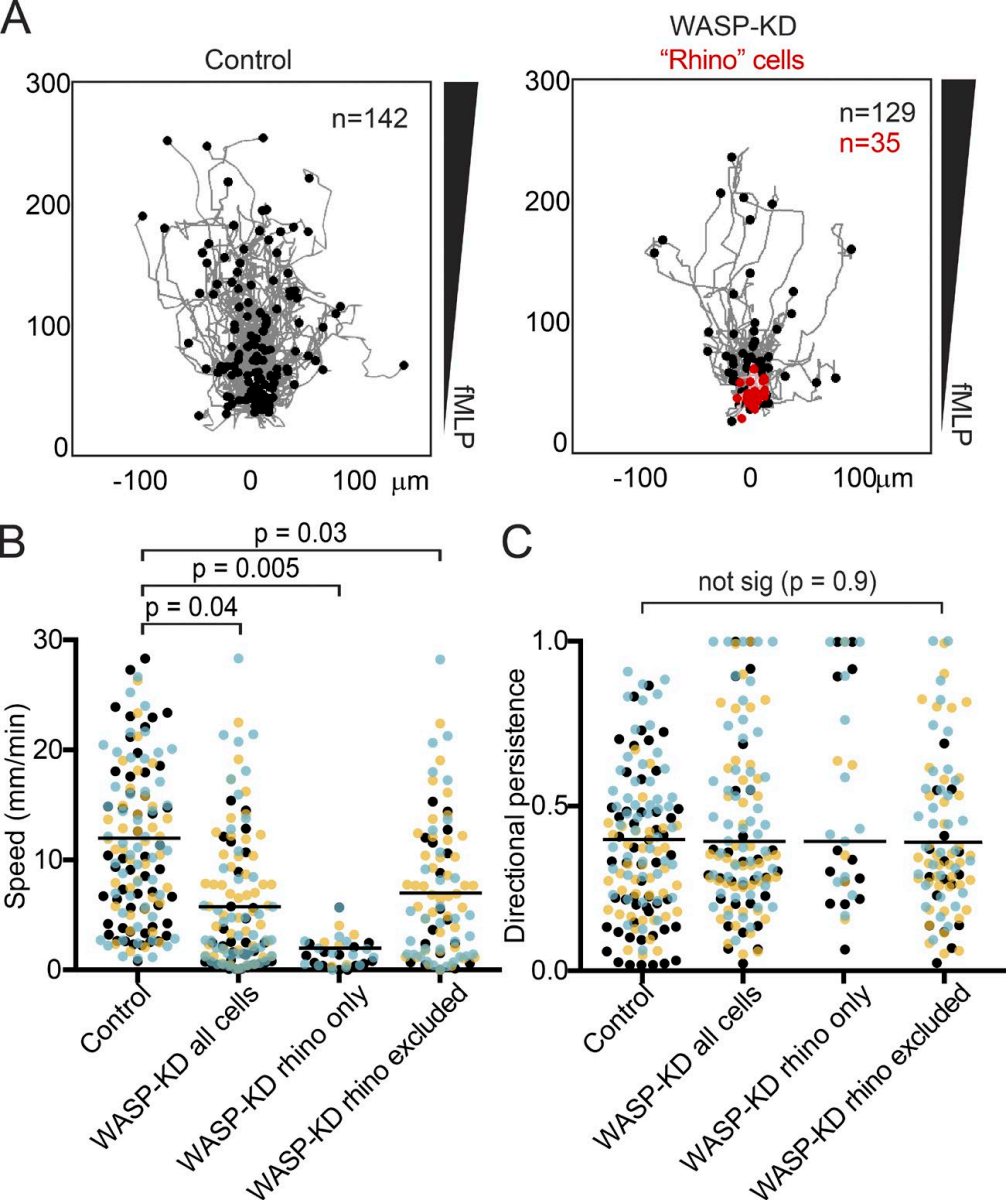
**WASP is crucial for neutrophil motility.** (A) Worm plots showing the tracks of cells migrating up an fMLP chemoattractant gradient. Control cells are on the left, and WASP-KD cells are on the right, with cells exhibiting the rhino phenotype in red. Cells were imaged for 20 min and migration paths were overlaid, with time 0 at (0,0). The endpoint of each cell’s path is shown with a dot. See also Video 2. (B) Depletion of WASP protein leads to reduced cell speed. The mean instantaneous speed for each cell in A is plotted as a dot color-coded by biological replicate to highlight the consistency from experiment to experiment. (C) Reduction of WASP protein leads to no significant change in directional persistence (the ratio of the Euclidean distance to the accumulated distance) of cells tracked in A. Means of the three replicates are displayed as horizontal lines; p-values were obtained from two-tailed paired *t* tests.

To determine the effect of WASP depletion on cell locomotion, we imaged HL-60 cells migrating through a chemoattractant gradient in a 5-µm-tall glass chamber (Millius and Weiner, 2010). Tracking individual cells revealed a severe migration defect in WASP-KD cells (reported means ± SD of three biological replicates): although control cells moved at 12 ± 0.8 µm/min, WASP-KD cells averaged 5.5 ± 1.5 µm/min, and cells with rhino protrusions were almost completely immotile, moving at 1.7 ± 0.4 µm/min (Fig. 4, A and B; and Video 2). This motility defect was not limited to the rhino cells: when these cells were excluded from the analysis, we still observed a significantly reduced speed (6.7 ± 0.8 µm/min) compared with control cells (Fig. 4 B). We did not observe an effect of WASP depletion on directional persistence (Fig. 4 C).

Because the chamber we used to measure directional migration was not precoated with fibronectin (or any other specific molecule), we doubt this migration defect was caused by an integrin-mediated adhesion defect. We confirmed this by directly testing adhesion to fibronectin-coated surfaces and found no significant difference between WASP-KD and control cells (Fig. S3 A). We conclude that HL-60 cells use WASP along with SCAR (Weiner et al., 2006) for normal pseudopod formation and efficient α-motility.

### WASP and SCAR genes predict pseudopod formation by chytrid fungi

A potential exception to the tight correlation between actin-rich pseudopods and the genomic retention of WASP and SCAR were two deeply branching and little-studied species of fungi: the chytrids *A. macrogynus* and Bd. These chytrid species contain genes encoding both WASP and SCAR but have not been reported in the literature to migrate using pseudopods. We were, however, able to find references to pseudopod formation by unsequenced infectious species related to *A. macrogynus* (*Catenaria anguillulae*), which may use these structures for motility across the surface of its target host (Deacon and Saxena, 1997; Gleason and Lilje, 2009). However, because chytrid fungi are not a monophyletic group but rather comprise multiple deeply branching clades that are estimated to have diverged ∼800 million years ago (James et al., 2006; Stajich et al., 2009), one cannot assume that distantly related species share this capacity. Therefore, we used Bd as a predictive test of our hypothesis that WASP and SCAR genes represent a marker for α-motility.

Like other species of chytrid fungi, the lifecycle of Bd has two stages: a large (10–40 µm) reproductive zoosporangium, which releases a host of small (3–5 µm), motile, and flagellated zoospore cells (Longcore et al., 1999; Berger et al., 2005). These infectious zoospores can form cysts beneath the skin of an amphibian host that develop into new zoosporangia to complete the life cycle (Berger et al., 2005). We searched for α-motility in Bd zoospores because, unlike the sessile cyst and zoosporangium, these free-swimming flagellates lack a cell wall and have been reported to assume nonuniform shapes with dense cytoplasmic extensions (Longcore et al., 1999).

To restrict the fast-swimming Bd zoospores to the imaging plane, we adhered zoospores to concanavalin A–coated glass. In initial experiments, we observed only a small fraction (<1%) of zoospores forming pseudopodlike protrusions. The rarity of pseudopod-forming cells made us suspect that α-motility might only occur during a short phase of the life cycle. We therefore enriched for cells of the same age by washing zoosporangia to remove previously released zoospores and collecting flagellates released during the subsequent 2 h.

**Figure 5.**
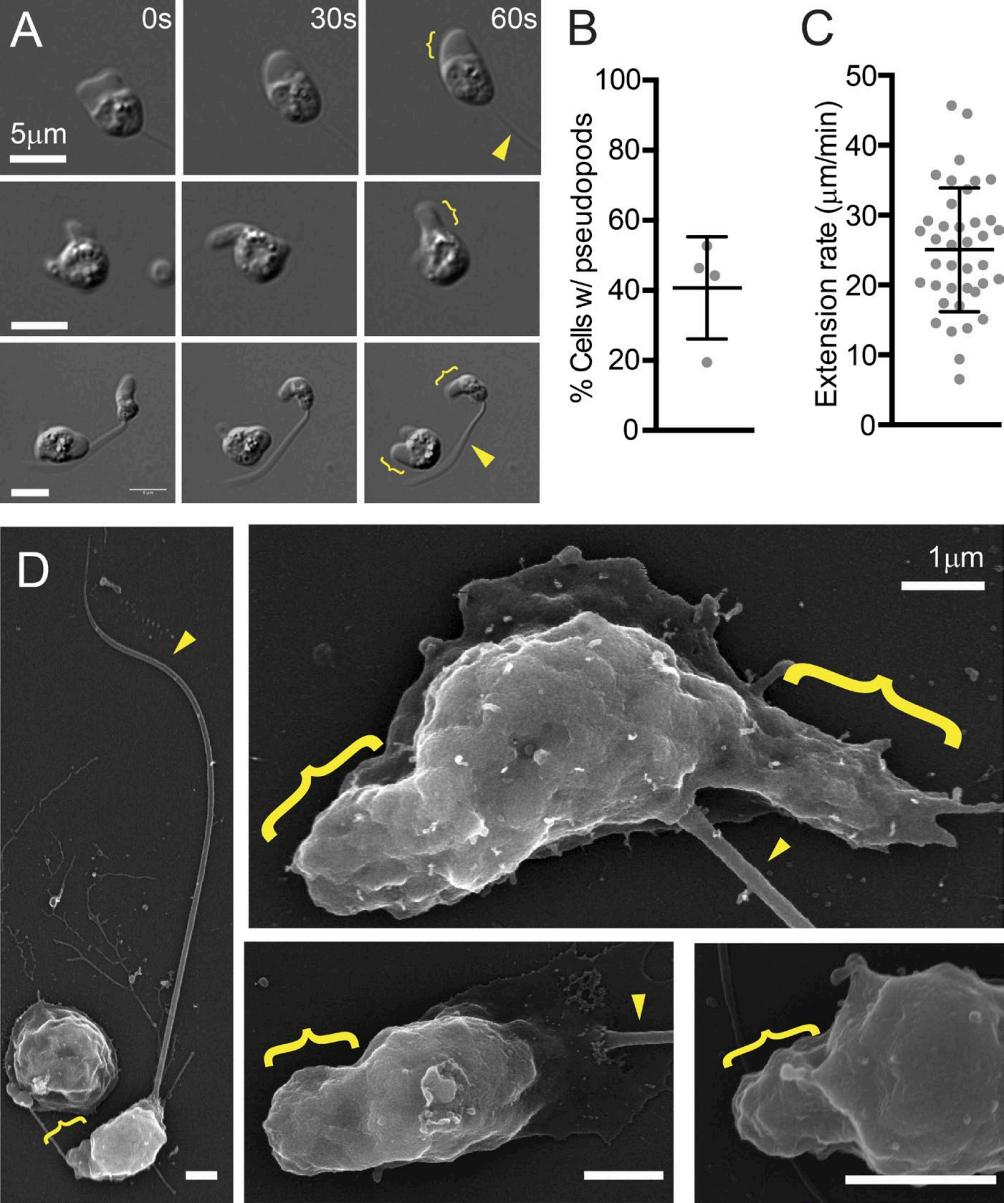
**Genomic retention of both WASP and SCAR correctly predicts pseudopod formation by the infectious chytrid fungus Bd.** (A) Time lapse showing examples of dynamic pseudopods from chytrid cells with (top) and without a flagellum (middle) or one cell of each (bottom). See also Video 3. (B) Percentage of cells with pseudopods within the first 6 h after release from zoosporangia. The mean and SD (bars) of four biological replicates (dots) is shown, with 3,782 cells total. (C) Pseudopod extension rates. The means and SD (bars) of the individual values (dots) combined from three biological replicates is shown. (D) Scanning electron micrographs of fixed chytrid zoospores. Brackets denote pseudopods and arrowheads denote flagella. See also Fig. S4 B for more examples.

During the first 6 h after release from the zoosporangium, ∼40% of zoospores created dynamic pseudopodlike protrusions (Fig. 5, A and B; Fig. S4 A; and Video 3) that extended from the cell body at a rate of 25 ± 9 µm/min (Fig. 5 C), consistent with speeds expected for pseudopods (Chodniewicz and Zhelev, 2003; Zhelev et al., 2004). Unlike blebs, these cellular protrusions were not spherical but were irregularly shaped and amorphous, similar to the actin-rich pseudopods of amoebae and neutrophils.

To ensure that these crawling cells were not contaminating organisms, we obtained Bd cultures from three different laboratories and observed similar pseudopods in each.

To better investigate the morphology of these tiny pseudopods, we performed scanning electron microscopy on fixed cells (Figs. 5 D and S4 B) and observed a similar proportion of flagellated zoospores with one or more thick protrusions. Each protrusion was ∼1 µm long and 1 µm wide, and many appeared to be composed of multiple discrete terraces (Figs. 5 D and S4 B).

### Chytrid pseudopods contain actin and require Arp2/3 activity

**Figure 6.**
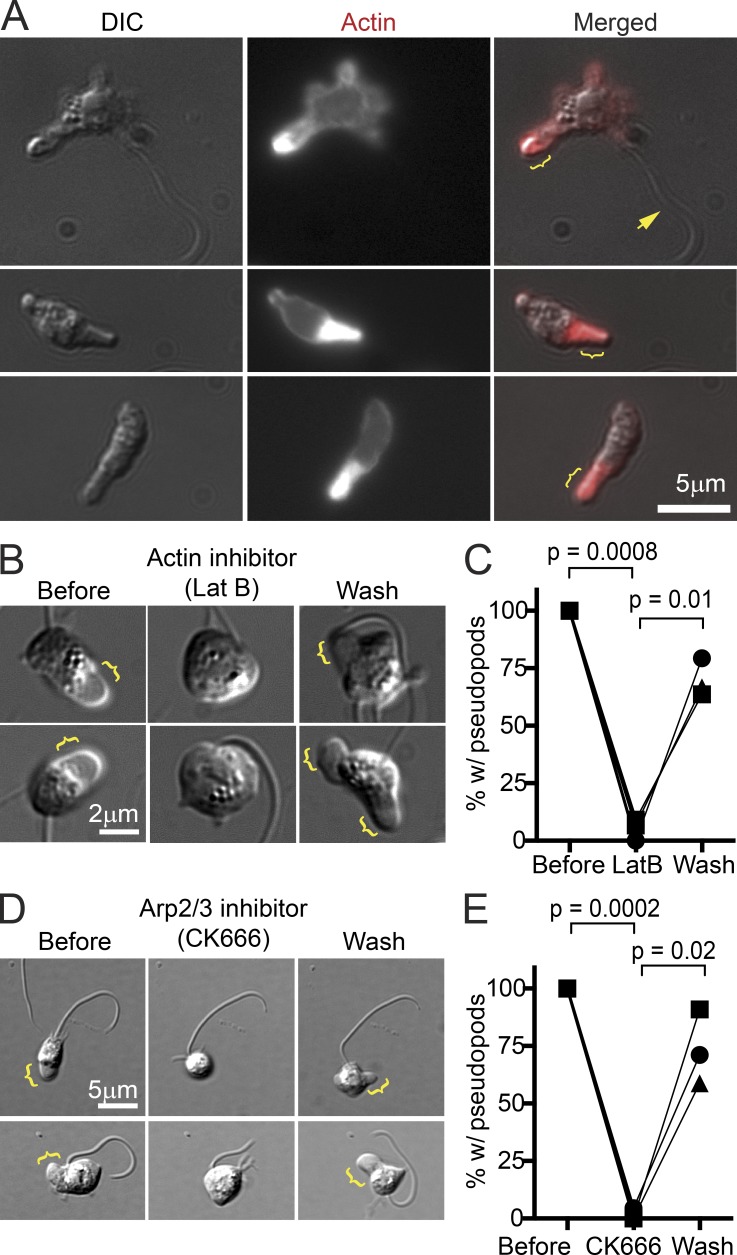
**Chytrid pseudopods are actin filled and require both actin polymerization and Arp2/3 activity.** (A) Fixed chytrid cells with and without a flagellum (arrow). Staining with fluorescent phalloidin reveals a thin shell of cortical actin surrounding the cell body and a dense network of polymerized actin filling the pseudopods (brackets). DIC, differential interference contrast. (B) Two examples of chytrid cells with pseudopods that lose them when treated with 10 nm latrunculin B, an inhibitor of actin polymerization. Dynamic pseudopods (brackets) return after drug washout. (C) Quantification of reversible inhibition of pseudopods by latrunculin B (Lat B). Only cells that were making pseudopods before treatment and that were not washed away during the experiment were counted. (D) Two examples of chytrid cells with pseudopods that lose them when treated with 10 µm CK-666, an inhibitor of Arp2/3 activity. Pseudopods return after drug washout. (E) Quantification of reversible inhibition of pseudopods by CK-666. Only cells that were making pseudopods before treatment and that were not washed away during the experiment were counted. Symbols are means from three biological replicates, each with at least 29 (C) or 17 (E) cells. P-values were obtained from two-tailed paired *t* tests.

Using our assay to image chytrid zoospores, we next investigated whether extension of Bd pseudopods is driven by assembly of branched actin networks as in other cells that crawl using α-motility. We first fixed the cells to preserve the actin cytoskeleton and then stained them with fluorescent phalloidin, revealing a thin shell of cortical actin surrounding the cell body and a dense network of filamentous actin filling the pseudopod (Fig. 6 A).

To test whether actin-filled chytrid pseudopods required actin polymerization, we treated zoospores with latrunculin, a small molecule that sequesters actin monomers and inhibits the growth of actin polymers. Within minutes of adding 10 nM latrunculin B, nearly all pseudopods ceased growing and/or disappeared (Fig. 6, B and C). This effect was reversed within 1 h after removing the drug.

To determine whether assembly of the pseudopodial actin network required the nucleation and branching activity of the Arp2/3 complex, we incubated zoospores with CK-666, a small molecule that inhibits actin nucleation by mammalian and fungal Arp2/3 complexes (Nolen et al., 2009). Addition of 10 µM CK-666 reduced the number of cells with active protrusions by nearly 100%, an effect that was reversed by washing out the drug (Fig. 6, D and E). These experiments reveal that protrusion of Bd pseudopods requires Arp2/3-dependent actin assembly.

### Chytrid zoospores use pseudopods for α-motility

**Figure 7.**
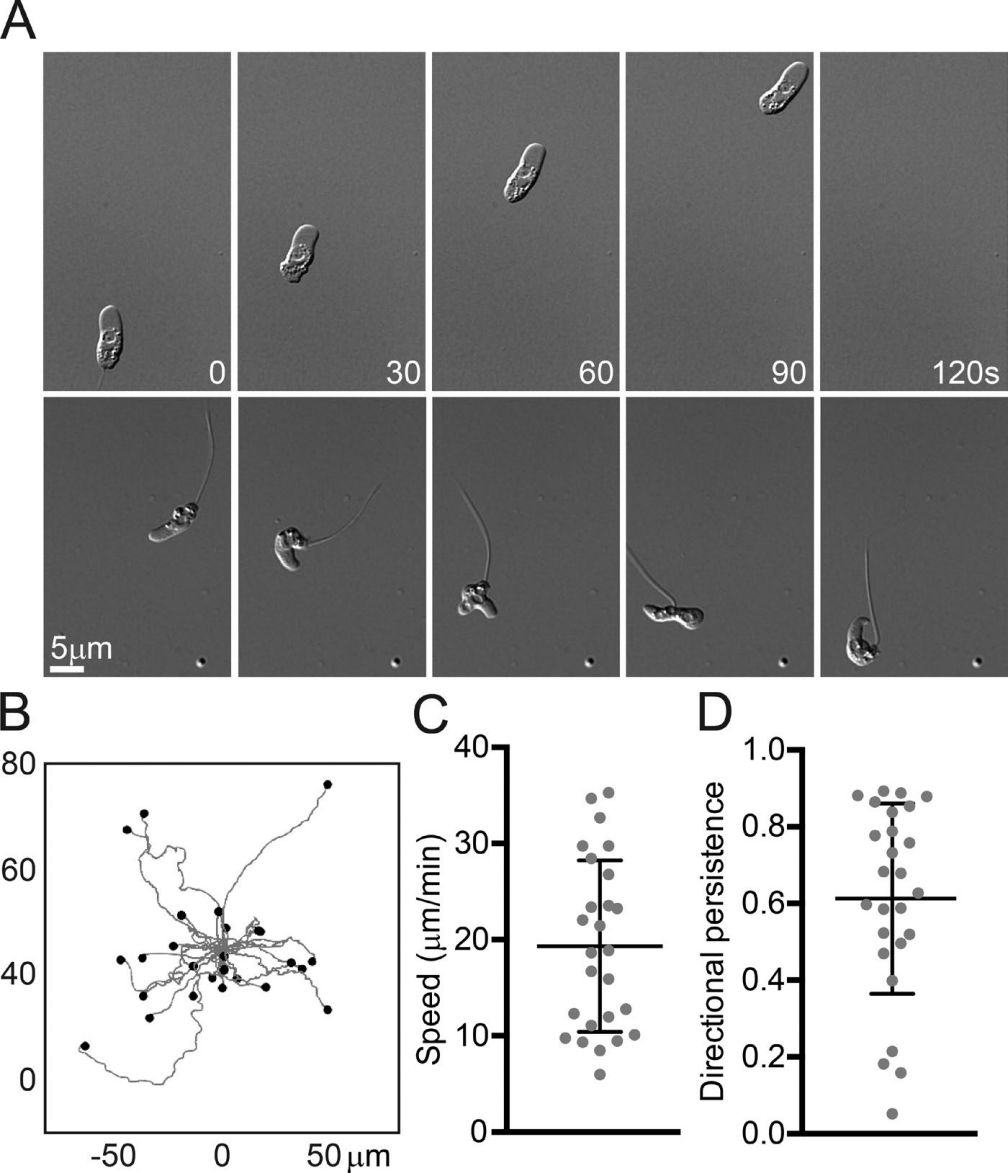
**Genomic retention of both WASP and SCAR correctly predicts α-motility in the infectious chytrid fungus Bd.** (A) Example chytrid zoospores with and without a flagellum migrating when confined between 1-µm spaced glass coverslips. See also Video 4. (B) Worm plots showing the tracks of 26 migrating chytrid zoospores with migration paths overlaid and with time 0 at (0,0). The endpoint of each cell’s path is shown with a dot. Only those cells obviously migrating were tracked, and cells were tracked for the duration of their movement. (C) Mean instantaneous speed of cells tracked in B. (D) Directional persistence (the ratio of the Euclidean distance to the accumulated distance) of cells tracked in B. Means and SD (bars) of the individual values (dots) combined from three biological replicates are shown.

Although pseudopod-forming Bd cells adhered tightly to glass surfaces coated with concanavalin A, they were not able to move or swim away from the site of initial attachment, and other coatings did not promote any form of attachment (including collagen, fibronectin, and human keratin; not depicted). Several types of animal cells are known to migrate without specific molecular adhesions in confined environments (Lämmermann et al., 2008; Liu et al., 2015; Ruprecht et al., 2015). To test whether Bd zoospores might also be capable of migration in confined environments, we sandwiched cells between two uncoated glass coverslips held apart by 1-µm-diameter glass microspheres and observed rapidly migrating cells (Fig. 7 and Video 4). Obviously migrating cells had a mean instantaneous speed of 19 ± 9 µm/min, with individual cells averaging speeds >30 µm/min (Fig. 7 C), consistent with the measured rates of pseudopod extension (Fig. 5 C). The trajectories of these cells appeared fairly straight (Fig. 7 B), with a mean directional persistence of 0.61 ± 0.25 (Fig. 7 D).

Some pseudopod-forming zoospores retained flagella, whereas other cells had clearly lost or resorbed their flagella and strongly resembled free-living amoebae (Fig. 7 A and Videos 3 and 4). We also observed cells switching from crawling to flagellar motility and vice versa as well as cells rapidly retracting their flagellar axonemes into the cell body (Video 5).

## Discussion

Our results reveal that across eukaryotic phyla, cells that construct actin-rich pseudopods and undergo fast, low-adhesion crawling have retained the Arp2/3 complex as well as two distinct activators of their actin nucleation activity: WASP and SCAR/WAVE. This finding is well supported by a recent paper implicating both WASP and SCAR in *Caenorhabditis elegans* neuroblast cell migration (Zhu et al., 2016). In that system, the phenotype of SCAR mutants is enhanced by the loss of WASP, and both WASP and SCAR are found at the leading edge of migrating neuroblasts in vivo. Our hypothesis is also consistent with a study of myoblast cell fusion events during muscle formation: like pseudopods, myoblast protrusions are an actin-filled force generating machines that require both WASP and SCAR (Sens et al., 2010).

**Figure 8.**
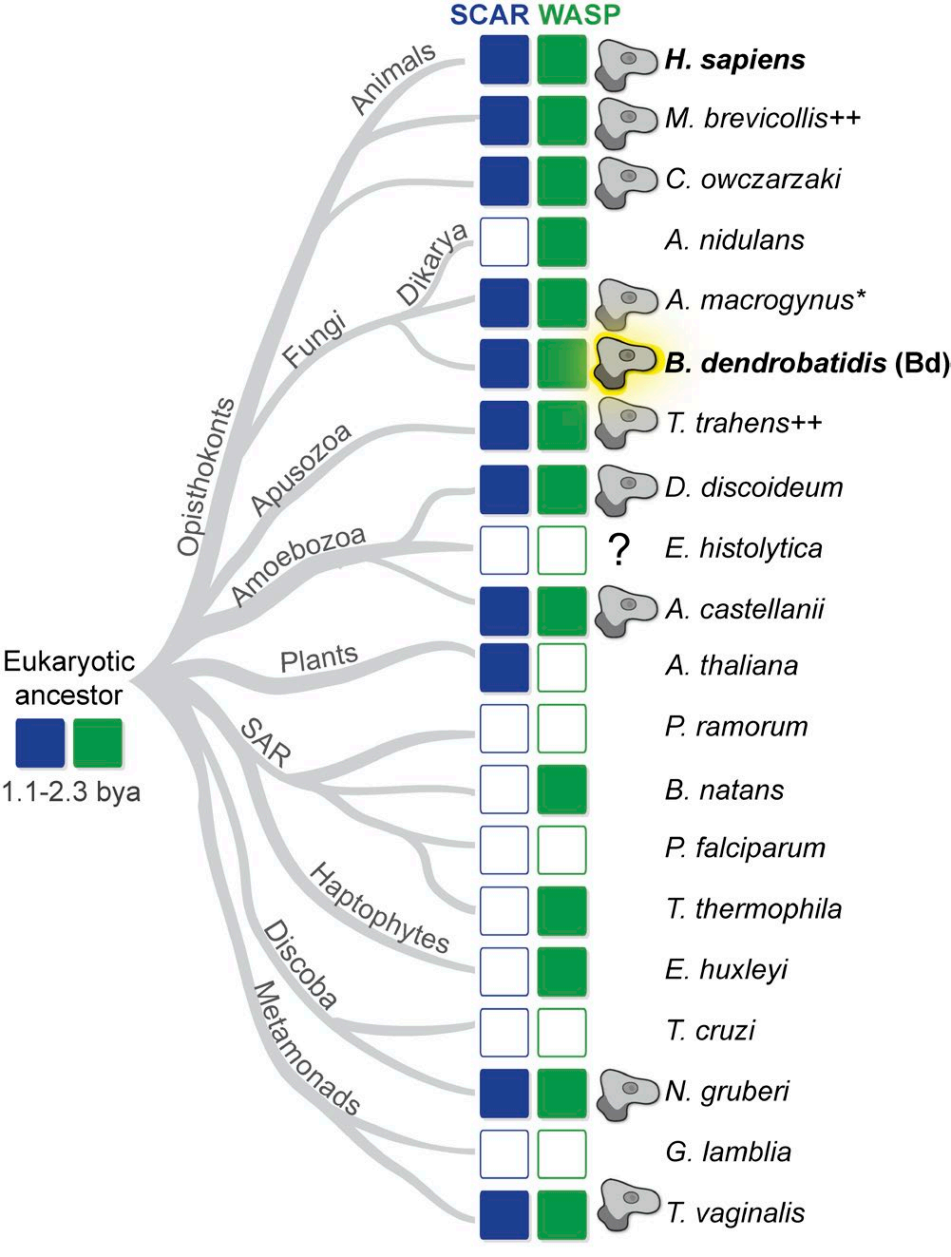
**Only organisms that make pseudopods retain both WASP and SCAR genes.** Diagram showing the relationships of extant eukaryotes (based on a study by Fritz-Laylin et al., 2010) with the presence or absence of SCAR (blue) and WASP (green) genes from complete genome sequences as described previously (Kollmar et al., 2012). Each representative organism whose genome was used for the analysis is listed to the right. For groups with similar morphological and sequence patterns, a single species is used. For example, there is no known plant species that forms pseudopods or retains the WASP gene, so only a single species is shown (*Arabidopsis thaliana*); similarly, *Aspergillus nidulans* represents all dikarya. See Kollmar et al. (2012) for additional sequence information. An amoeba glyph indicates organisms that build pseudopods. Outlined rectangles indicate a lack of an identifiable gene. See Table 1 for citations and full species names. *, Although we were not able to find a reference to pseudopod formation in *A. macrogynus*, a relative (*C. anguillulae)* does assemble pseudopods used for motility (Deacon and Saxena, 1997; Gleason and Lilje, 2009). Because of this and the conservation of both WASP and SCAR in Bd (highlighted in bold), we correctly predicted this species is also capable of pseudopod formation. ^++^, These species form pseudopods for feeding rather than motility. The question mark indicates uncertainty regarding the structure of the protrusions for phagocytosis in *E. histolytica* (see the Evolutionary retention of both WASP and SCAR correlates with pseudopod formation section). The time of divergence of extant eukaryotic groups has been estimated to be 1.1–2.3 billion years ago (bya; Chernikova et al., 2011; Parfrey et al., 2011; Knoll, 2014) and has been predicted to have possessed both WASP and SCAR gene families (Kollmar et al., 2012) and therefore may have built pseudopods.

Organisms without the capacity to crawl using pseudopods turn out to have lost one or both of these nucleation-promoting factors (Fig. 8 and Table 1). The presence of genes encoding both WASP and SCAR, therefore, provides a molecular correlate for a suite of behaviors that we call “α-motility.” The conservation and phylogeny of WASP and SCAR indicate that both were present in a common ancestor of living eukaryotes (Veltman and Insall, 2010; Kollmar et al., 2012). The power of the coconservation of WASP and SCAR as a genomic marker with the ability to identify cryptic pseudopod-forming organisms—together with cell biology evidence that both WASP and SCAR are required for α-motility in well-studied organisms—argues that this widespread behavior arose from a single, ancient origin. It is formally possible that α-motility did not have a single evolutionary origin, but that scenario would require both WASP and SCAR to be coopted together for pseudopod assembly multiple times during eukaryotic evolution. Because WASP and SCAR are only two of a large number of Arp2/3 activators (Rottner et al., 2010), we have no reason to believe that motility would repeatedly converge on these two in particular.

Metazoans and fungi, together with a handful of protists, form a major clade known as the “opisthokonts” (Fig. 8). Our identification of α-motility in a fungal species argues that the ancestor of all the opisthokonts was capable of fast pseudopod-associated crawling and that multicellular fungi represent lineages that have lost α-motility (Fritz-Laylin et al., 2010). Because cells of multicellular fungi are always encased in rigid cell walls that would block pseudopods, selection pressure to preserve gene networks specific to pseudopod formation was relieved, and the genes unique to this behavior were subsequently lost. The fungi would therefore represent a large eukaryotic lineage from which crawling motility has almost completely disappeared.

Images from an earlier study revealed individual Bd zoospores with irregular shapes and cytoplasmic extensions (Longcore et al., 1999). Actin-driven pseudopod formation and cell motility, however, were not previously described in Bd cells, in part because this species was discovered quite recently (Longcore et al., 1999), and relatively few studies have been devoted to its cell biology. In addition, Bd zoospores are quite small (<5 µm) and highly motile, so visualizing their tiny (∼1 µm) pseudopods requires physical confinement and high-resolution microscopy. Finally, because only recently released zoospores crawl, synchronization of cell cultures was crucial. These advances not only enabled us to observe α-motility but also revealed Bd zoospores retracting their flagella by coiling the entire axoneme into the cell body in <1 s (Video 5), a process that has been observed to take minutes in other chytrid species (Koch, 1968).

The α-motility of Bd fills an important gap in our understanding of the life cycle of this pathogen. As proposed for other chytrid species (Gleason and Lilje, 2009), Bd zoospores may use pseudopods during the initial stages of their interaction with a host either to move across epithelia or to crawl between epithelial cells and invade the underlying stroma. Alternatively, our observation that newly hatched zoospores make more pseudopods suggests that Bd may rely on α-motility to crawl along or within the epithelial surface to uninfected tissues or to exit the host.

Imaging chytrid zoospores provided key evidence for the involvement of WASP and SCAR in a conserved mode of cell migration, but further exploration of WASP and SCAR function in Bd is hampered by several factors. First, the zoospores are small enough to pose challenges to live-cell imaging. Second, the absence of genetic tools makes it impossible to fluorescently label or deplete proteins in live zoospores. Finally, the lack of potent and specific inhibitors of WASP and SCAR (Guerriero and Weisz, 2007; Bompard et al., 2008) precludes chemical disruption of their activity.

Several protein families are known to activate the Arp2/3 complex, including WASP, SCAR, WASH, JMY, and WHAMM (Rottner et al., 2010). A conventional explanation for the multiplicity of Arp2/3 activators is that each promotes the construction of an actin network with a unique cellular function and/or location. However, the evolutionary connection between WASP, SCAR, and pseudopod formation suggests that nucleation-promoting factors can work together, in this case to drive the explosive actin polymerization required for α-motility. Indeed, we find that WASP and SCAR colocalize at the leading edge of crawling neutrophils, and WASP depletion results in aberrant pseudopods and reduced motility similar to reported effects of SCAR depletion (Weiner et al., 2006). But why have multiple distinct Arp2/3 activators instead of simply increasing the concentration of one of them? The answer may lie in the positive feedback that drives the explosive actin polymerization required for α-motility, but which can also result in spurious pseudopod formation (Chung et al., 2000). We propose that the use of two distinct activation systems, WASP and SCAR, reduces the probability of errant pseudopod formation by using both activators to raise Arp2/3 activity above the threshold required for robust pseudopod formation.

Under this coincidence detection model, an occasional spike in the local activity of either WASP or SCAR may be sufficient to trigger pseudopod formation, but activation of both should be more efficient at driving explosive actin assembly. This fits our observation that WASP depletion results in a subset of cells capable of pseudopod formation but with less polymerized actin (Fig. 3, C and D) and reduced migration speeds (Fig. 4 B) and explains previous studies showing the incomplete penetrance of WASP and SCAR phenotypes (Blagg et al., 2003; Myers et al., 2005; Snapper et al., 2005; Weiner et al., 2006; Veltman et al., 2012). This model may also explain the rhino phenotype: without the additional Arp2/3 activation provided by WASP, the resulting sparse actin networks may collapse and coalesce into the observed horn-shaped structures. This idea is supported by studies of the upstream activators of WASP (Cdc42) and SCAR (Rac) that indicate that Rac mediates a positive feedback loop required for leading-edge formation but that the stability of the resulting protrusion requires Cdc42 (Srinivasan et al., 2003; Stradal and Scita, 2006).

This model also clears up confusion in the field regarding WASP’s role in cell migration. Our results are supported by papers showing that blood cells rely on WASP for efficient cell migration (Binks et al., 1998; Zicha et al., 1998; Anderson et al., 2003; Snapper et al., 2005; Zhang et al., 2006; Blundell et al., 2008; Dovas et al., 2009; Kumar et al., 2012; Worth et al., 2013) and others suggesting that WASP plays a direct role in protrusion formation, including pseudopods (Badolato et al., 1998; Burns et al., 2001; Jones et al., 2002, 2013; Shi et al., 2009; Ishihara et al., 2012). However, these data have been overshadowed by studies showing that fibroblasts do not require N-WASP for filopodia or sheet-like surface-adhered lamellipodia (Lommel et al., 2001; Snapper et al., 2001; Sarmiento et al., 2008). Such papers have been cited as proof that all WASP family proteins are dispensable for protrusions in general (Small and Rottner, 2010). Such generalizations depend on two assumptions: that N-WASP and WASP have the same molecular function and that the adherent motility of fibroblasts and α-motility use the same molecular pathways. However, a recent molecular replacement study showed that WASP and the ubiquitously expressed N-WASP have different functions and cannot compensate for each other (Jain and Thanabalu, 2015). Furthermore, when one considers the large body of mammalian WASP literature in the light of distinct modes of motility, a simple pattern emerges: cell types that do not natively express WASP do not make pseudopods (although they may make surface-bound lamellipodia, linear filopodia, or adhesive structures called podosomes); WASP is only expressed in blood cells, and these cells use WASP for pseudopod-based migration (see Tables S1 and S2 for an annotated summary of WASP/N-WASP literature). The predominant view that WASP is not involved in cell migration demonstrates the peril of assuming that insights based on adhesion-dependent cell motility apply to other modes of cell crawling.

In addition to motility, Arp2/3 activators have been shown to play roles in other cellular processes, including endocytosis (Naqvi et al., 1998; Merrifield et al., 2004; Benesch et al., 2005). The relationship between cell motility and endocytosis is complex and not completely understood (Traynor and Kay, 2007; Schiefermeier et al., 2011). Rapid pseudopod extension requires not only a large quantity of actin polymerization (Weiner et al., 2006) but also increases membrane tension (Diz-Muñoz et al., 2016), both of which counteract efficient clathrin- and actin-mediated endocytosis (Boulant et al., 2011). Despite this apparent dichotomy between protrusion formation and endocytosis, both SCAR and WASP protein families have been shown to interact with endocytosis pathways (Badour et al., 2007; Gautier et al., 2011). Accordingly, we found that WASP-deficient HL-60 cells maintained normal receptor internalization and recycling (Fig. S3 E) until pseudopod activity was activated by differentiation into neutrophils. After differentiation, in addition to being defective in building pseudopods, WASP-KD cells exhibited increased endocytosis and receptor recycling (Fig. S3, B–D). This is consistent with the idea that actin-mediated endocytosis is more efficient when cells are not making pseudopods.

Although a large number of eukaryotes make pseudopods (Fig. 8 and Table 1), only two lineages are currently genetically tractable: animals and dictyostelids. Studies in both confirm that pseudopod formation involves both WASP (Badolato et al., 1998; Burns et al., 2001; Jones et al., 2002, 2013; Shi et al., 2009; Ishihara et al., 2012) and SCAR (Miki et al., 1998; Steffen et al., 2004; Weiner et al., 2006; Veltman et al., 2012), in contrast to some animal cell types that may only require SCAR for lamellipodia-based migration (Snapper et al., 2001; Bryce et al., 2005; Misra et al., 2007; Sarmiento et al., 2008). With our discovery of α-motility in the fungus Bd, we conclude that both proteins have been conserved together to facilitate this evolutionarily ancient mode of cell motility.

## Materials and methods

### Antibodies and Western blotting

Rabbit anti-WASP antibody (sc-8353) was from Santa Cruz Biotechnology, Inc., as was the goat anti-WAVE2 (sc-10394). Mouse anti-tubulin (DM1A) was from Sigma-Aldrich, and mouse anti-actin (JLA20) was from EMD Millipore. Western blotting was conducted using standard protocols and HRP-conjugated secondary antibodies (Jackson ImmunoResearch Laboratories, Inc.).

### Generation of HL-60 cell lines

HL-60 lines were derived from CCL-240 (ATCC) and were grown in RPMI 1640 medium supplemented with 15% FBS, 25 mM Hepes, and 2.0 g/liter NaHCO_3_ and were grown at 37°C and 5% CO_2_. WASP-KD was achieved using Sigma-Aldrich’s Mission Control shRNA vector (TRCN0000029819; hairpin sequence 5′-CCGGCGAGACCTCTAAACTTATCTACTCGAGTAGATAAGTTTAGAGGTCTCGTTTTT-3′) with corresponding control vector expressing anti-GFP shRNA (SHC005; hairpin sequence 5′-CCGGCGTGATCTTCACCGACAAGATCTCGAGATCTTGTCGGTGAAGATCTTTTT-3′). Lentivirus was produced in HEK293T grown in six-well plates and transfected with equal amounts of the lentiviral backbone vector (either protein expression vector derived from pHRSIN-CSGW [Demaison et al., 2002] or shRNA expression vectors described in the previous sentence), pCMVΔ8.91 (encoding essential packaging genes), and pMD2.G (encoding VSV-G gene to pseudotype virus). pHRSIN-CSGW and packaging vectors were obtained from R. Vale (University of California, San Francisco, San Francisco, CA). After 48 h, the supernatant from each well was removed, centrifuged at 14,000 *g* for 5 min to remove debris, and then incubated with ∼10^6^ HL-60 cells suspended in 1 ml complete RPMI for 5–12 h. Fresh medium was then added, and the cells were recovered for 3 d to allow for target protein or shRNA expression. TagRFPt-WASP fusion was cloned by first swapping out eGFP for TagRFP-T (Shaner et al., 2008) in the pHRSIN-CSGW by PCR amplifying TagRFP-T with 5′-CCCGGGATCCACCGGTCGCCACCATGGTGTCTAAGGGCGAAGAGCTGATTAAGG-3′ and 5′-GAGTCGCGGCCGCTTTAACTAGTCCCGCTGCCCTTGTACAGCTCGTCCATGCCATTAAGTTTGTGCCCC-3′ primers and then cloning the resulting PCR product into pHRSIN-CSGW, using NotI and BamHI to produce the pHR-TagRFP-T vector. Then, the WASP open reading frame was PCR amplified from cDNA (NCBI accession number BC012738) using 5′-GCACTAGTATGAGTGGGGGCCCAATGGGAGGAA-3′ and 5′-AAGCGGCCGCTCAGTCATCCCATTCATCATCTTCATCTTCA-3′ primers and then cloned into the pHR-TAGRFP-T backbone using NotI and SpeI to result in a single open reading frame containing TagRFPt, a flexible linker (amino acids GSGTS) followed by full-length WASP. The WASP shRNA rescue vector was cloned by inserting a P2A cleavage site (Kim et al., 2011) between the linker and a WASP open reading frame edited with site-directed mutagenesis to contain three silent mutations within the shRNA-targeting region (5′-CGAGACCTCTAAACTTATCTA-3′ was changed to 5′-CGAaACCTCTAAgCTcATCTA-3′; silent mutations in lowercase). A corresponding control vector was designed to express TagRFPt with the flexible linker but no portion of WASP. The Hem1-YFP line (R6) was previously described (Weiner et al., 2007) and was developed by selecting HL-60 cells expressing both an shRNA-targeting native Hem1 and a YFP-tagged version of Hem-1 allowing fluorescence imaging of the SCAR complex in HL-60 cells. shRNA lines were selected by puromycin (1 µg/ml for at least 1 wk) and fluorescent cell lines by FACS. HL-60 cells were differentiated by treatment with 1.3% DMSO for 5 d.

### Cytometry

FACS analysis was performed on a FACSCalibur analyzer (BD), Data were analyzed with FlowJo software (Tree Star), and dead cells were gated out using forward and side scatter for all analyses. A FACS Aria II was used for sorting. All FACS analysis was performed at the University of California, San Francisco, Laboratory for Cell Analysis.

### Imaging

EZ-TAXIScan (Effector Cell Institute) analysis of HL-60 cell migration between glass surfaces was conducted as previously described (Millius and Weiner, 2010), and cell migration analyzed using the Chemotaxis and Migration Tool (Ibidi). Fixed HL-60 cells were imaged with a 100× 1.40 NA oil Plan Apo objective on a motorized inverted microscope (T*i*-E; Nikon) equipped with a spinning disk (CSU22; Yokogawa Electric Corporation) and an electron-multiplying charge-coupled device camera (Evolve; Photometrics). Live TIRF images were acquired by plating HL-60 cells on cover glass cleaned by a 30 min incubation in 3 M NaOH and then four washes with PBS, pH 7.2, coated for 30 min with 100 µg/ml bovine fibronectin (F4759; Sigma-Aldrich) resuspended in PBS. TIRF microscopy images were acquired on an inverted microscope (TE2000; Nikon) equipped with a 60× or 100× 1.49 NA oil Apo TIRF objective and an electron-multiplying charge-coupled device (iXon+; Andor Technology) using previously described imaging conditions (Weiner et al., 2007). In brief, differentiated HL-60 cells were plated on fibronectin-coated coverslips in modified HBSS supplemented with 0.2% serum albumin and imaged with 100-ms exposures every 1–2 s. Fixed chytrid cells were imaged using an inverted T*i*-E microscope equipped with a spinning-disk confocal system with 33-µm pinholes and a 1.8× tube lens (Spectral Diskovery), a 60× 1.49 NA Apo TIRF objective (Nikon), and a complementary metal oxide semiconductor camera (Zyla 4.2; Andor Technology). Differential interference contrast microscopy was performed on a T*i*-E inverted microscope with a light-emitting diode illuminator (TLED; Sutter Instrument) and a 100× 1.49 NA Apo TIRF objective. Images were acquired on a complementary metal oxide semiconductor camera. All microscopy hardware was controlled with Micro-Manager software (Edelstein et al., 2010). Image analysis was performed with the ImageJ bundle Fiji (National Institutes of Health; Schindelin et al., 2012). All imaging was done at room temperature.

### Quantification of actin polymerization by flow cytometry

HL-60 cells were depolarized in serum-free medium supplemented with 2% low-endotoxin BSA (Sigma-Aldrich) for 1 h at 37°C and 5% CO_2_ before simulation with 20 nM fMLP for the indicated time. Cells were immediately fixed with 4% paraformaldehyde in cytoskeleton buffer on ice for 20 min, stained with PBS supplemented with 2% BSA, 0.1% Triton X-100, and 66 nM Alexa Fluor 488–conjugated phalloidin (A12379; Molecular Probes) for 20 min, and then washed thrice with PBS supplemented with 0.1% Tween-20 before FACS analysis.

### Cell adhesion assay

Differentiated control and WASP-KD HL-60 cells were each stained with either green or blue acetoxymethyl ester dyes (CellTrace Calcein green and blue; Thermo Fisher Scientific), and equal numbers were mixed and allowed to attach to fibronectin-coated cover glass–bottomed 96-well plates for 30 min at 37°C. One set of wells was gently washed three times with fresh media. 100 random locations within the well were immediately imaged, and the percentage of remaining cells was calculated and normalized to control unwashed wells.

### Transferrin uptake endocytosis assays

5 × 10^6^ differentiated HL-60 cells were washed twice with ice-cold serum-free growth medium (SF), transferred to 37°C for 5 min (to clear surface-bound transferrin), and chilled on ice for 1 min. An equal volume of cold SF supplemented with 100 µg/ml Alexa Fluor 488–conjugated transferrin (T-13342; Molecular Probes; McGraw and Subtil, 2001) was added and incubated on ice for 10 min. Cells were then washed twice with cold SF medium, transferred to 37°C for the indicated time period, washed twice with ice-cold acid buffer (8.76 g NaCl, 9.74 g 2-morpholin-4-ylethanesulfonic acid in 900 ml, pH to 4.0, and water to 1 liter), fixed in 4% paraformaldehyde in 1× PBS for 20 min, and washed twice more with ice-cold PBS before immediate FACS analysis.

### Motility of chytrid zoospores

The Bd strain JEL423 was obtained from J. Longcore (University of Maine, Orono, ME) and grown in 1% tryptone broth or on agar plates (1% tryptone and 2% agar) at 25°C. Before imaging, cultures were synchronized by either washing three times in 1% tryptone and harvesting zoospores 2 h later (for liquid cultures) or by flooding agar plates with ∼2 ml water (for agar plates) passed through a 40-µm filter (Falcon), were collected by centrifuging at 1,200 *g* for 5 min, and then were resuspended in Bonner’s Salts (Bonner, 1947). Cell motility was imaged by sandwiching cells between a number 1.5 glass coverslip and glass slide (cleaned by sonicating in pure water) separated using 1-µm glass microspheres (Bangs Laboratories). Coverslips and glass slides were sonicated in deionized water and dried. Cells were treated with either 10-µM CK-666 (Sigma-Aldrich) or 10-nM latrunculin B (Sigma-Aldrich) while being adhered to concanavalin A–coated glass. For visualization of polymerized actin: 400 µl fixation buffer (50 mM cacodylate buffer, pH 7.2) supplemented with 4% glutaraldehyde was added to 100 µl cells attached to a concanavalin A–coated coverslip and incubated for 20 min at 4°C. Samples were quenched with tetraborohydride, permeabilized with 0.1% Triton X-100, incubated for 20 min with Alexa Fluor 488–labeled phalloidin (Invitrogen), rinsed four times, and imaged described in the Imaging section. Samples for scanning electron microscopy were fixed as for visualization of polymerized actin, stained with osmium tetroxide, dehydrated, critical point dried, and Au/Pd sputter–coated according to standard protocols and then were imaged using a scanning electron microscope (S-5000; Hitachi) in the University of California, Berkeley, Electron Microscopy Laboratory.

### Online supplemental material 

Fig. S1 shows how WASP localizes to pseudopods of migrating neutrophils. Fig. S2 shows how WASP depletion in neutrophils leads to dynamic rhino protrusions. Fig. S3 shows how WASP is not required for adhesion or endocytosis by HL-60 cells. Fig. S4 shows additional examples of chytrid pseudopods. Video 1 shows TIRF microscopy of HL-60 cells. Video 2 shows chemotaxis of HL-60 cells. Video 3 shows time lapses of chytrid zoospores making pseudopods. Video 4 shows chytrid cells crawling. Video 5 shows an example of a chytrid zoospore retracting its flagellum. Tables S1 and S2 summarize the literature on the roles of WASP and N-WASP in protrusion formation and cell motility.

## Supplementary Material

Supplemental Materials (PDF)

Video 1

Video 2

Video 3

Video 4

Video 5

## References

[bib1] Anderson S.I., Behrendt B., Machesky L.M., Insall R.H., and Nash G.B. 2003 Linked regulation of motility and integrin function in activated migrating neutrophils revealed by interference in remodelling of the cytoskeleton. Cell Motil. Cytoskeleton. 54:135–146. 10.1002/cm.10091 12529859

[bib2] Axelrod D. 1981 Cell-substrate contacts illuminated by total internal reflection fluorescence. J. Cell Biol. 89:141–145. 10.1083/jcb.89.1.141 7014571 PMC2111781

[bib3] Babuta M., Mansuri M.S., Bhattacharya S., and Bhattacharya A. 2015 The *Entamoeba histolytica*, Arp2/3 complex is recruited to phagocytic cups through an atypical kinase EhAK1. PLoS Pathog. 11:e1005310 10.1371/journal.ppat.1005310 26646565 PMC4672914

[bib4] Badolato R., Sozzani S., Malacarne F., Bresciani S., Fiorini M., Borsatti A., Albertini A., Mantovani A., Ugazio A.G., and Notarangelo L.D. 1998 Monocytes from Wiskott-Aldrich patients display reduced chemotaxis and lack of cell polarization in response to monocyte chemoattractant protein-1 and formyl-methionyl-leucyl-phenylalanine. J. Immunol. 161:1026–1033. 9670984

[bib5] Badour K., McGavin M.K.H., Zhang J., Freeman S., Vieira C., Filipp D., Julius M., Mills G.B., and Siminovitch K.A. 2007 Interaction of the Wiskott-Aldrich syndrome protein with sorting nexin 9 is required for CD28 endocytosis and cosignaling in T cells. Proc. Natl. Acad. Sci. USA. 104:1593–1598. 10.1073/pnas.0610543104 17242350 PMC1785243

[bib6] Beltzner C.C., and Pollard T.D. 2004 Identification of functionally important residues of Arp2/3 complex by analysis of homology models from diverse species. J. Mol. Biol. 336:551–565. 10.1016/j.jmb.2003.12.017 14757065

[bib7] Benesch S., Polo S., Lai F.P.L., Anderson K.I., Stradal T.E.B., Wehland J., and Rottner K. 2005 N-WASP deficiency impairs EGF internalization and actin assembly at clathrin-coated pits. J. Cell Sci. 118:3103–3115. 10.1242/jcs.02444 15985465

[bib8] Berger L., Hyatt A.D., Speare R., and Longcore J.E. 2005 Life cycle stages of the amphibian chytrid *Batrachochytrium dendrobatidis*. Dis. Aquat. Organ. 68:51–63. 10.3354/dao068051 16465834

[bib9] Bergert M., Chandradoss S.D., Desai R.A., and Paluch E. 2012 Cell mechanics control rapid transitions between blebs and lamellipodia during migration. Proc. Natl. Acad. Sci. USA. 109:14434–14439. 10.1073/pnas.1207968109 22786929 PMC3437886

[bib10] Binks M., Jones G.E., Brickell P.M., Kinnon C., Katz D.R., and Thrasher A.J. 1998 Intrinsic dendritic cell abnormalities in Wiskott-Aldrich syndrome. Eur. J. Immunol. 28:3259–3267. 10.1002/(SICI)1521-4141(199810)28:10<3259::AID-IMMU3259>3.0.CO;2-B 9808195

[bib11] Blagg S.L., Stewart M., Sambles C., and Insall R.H. 2003 PIR121 regulates pseudopod dynamics and SCAR activity in *Dictyostelium*. Curr. Biol. 13:1480–1487. 10.1016/S0960-9822(03)00580-3 12956949

[bib12] Blundell M.P., Bouma G., Calle Y., Jones G.E., Kinnon C., and Thrasher A.J. 2008 Improvement of migratory defects in a murine model of Wiskott-Aldrich syndrome gene therapy. Mol. Ther. 16:836–844. 10.1038/mt.2008.43 18388921

[bib13] Bompard G., Rabeharivelo G., and Morin N. 2008 Inhibition of cytokinesis by wiskostatin does not rely on N-WASP/Arp2/3 complex pathway. BMC Cell Biol. 9:42 10.1186/1471-2121-9-42 18667055 PMC2527559

[bib14] Bonner J.T. 1947 Evidence for the formation of cell aggregates by chemotaxis in the development of the slime mold Dictyostelium discoideum. J. Exp. Zool. 106:1–26. 10.1002/jez.1401060102 20268085

[bib15] Boulant S., Kural C., Zeeh J.-C., Ubelmann F., and Kirchhausen T. 2011 Actin dynamics counteract membrane tension during clathrin-mediated endocytosis. Nat. Cell Biol. 13:1124–1131. 10.1038/ncb2307 21841790 PMC3167020

[bib16] Bowers B., and Korn E.D. 1968 The fine structure of *Acanthamoeba castellanii*. J. Cell Biol. 39:95–111. 10.1083/jcb.39.1.95 5678452 PMC2107510

[bib17] Bryce N.S., Clark E.S., Leysath J.L., Currie J.D., Webb D.J., and Weaver A.M. 2005 Cortactin promotes cell motility by enhancing lamellipodial persistence. Curr. Biol. 15:1276–1285. 10.1016/j.cub.2005.06.043 16051170

[bib18] Buenemann M., Levine H., Rappel W.-J., and Sander L.M. 2010 The role of cell contraction and adhesion in *dictyostelium* motility. Biophys. J. 99:50–58. 10.1016/j.bpj.2010.03.057 20655832 PMC2895335

[bib19] Burkel B.M., von Dassow G., and Bement W.M. 2007 Versatile fluorescent probes for actin filaments based on the actin-binding domain of utrophin. Cell Motil. Cytoskeleton. 64:822–832. 10.1002/cm.20226 17685442 PMC4364136

[bib20] Burns S., Thrasher A.J., Blundell M.P., Machesky L., and Jones G.E. 2001 Configuration of human dendritic cell cytoskeleton by Rho GTPases, the WAS protein, and differentiation. Blood. 98:1142–1149. 10.1182/blood.V98.4.1142 11493463

[bib21] Butler K.L., Ambravaneswaran V., Agrawal N., Bilodeau M., Toner M., Tompkins R.G., Fagan S., and Irimia D. 2010 Burn injury reduces neutrophil directional migration speed in microfluidic devices. PLoS One. 5:e11921 10.1371/journal.pone.0011921 20689600 PMC2912851

[bib22] Cavalier-Smith T., and Chao E.E. 2010 Phylogeny and evolution of Apusomonadida (protozoa: Apusozoa): New genera and species. Protist. 161:549–576. 10.1016/j.protis.2010.04.002 20537943

[bib23] Chernikova D., Motamedi S., Csürös M., Koonin E.V., and Rogozin I.B. 2011 A late origin of the extant eukaryotic diversity: divergence time estimates using rare genomic changes. Biol. Direct. 6:26 10.1186/1745-6150-6-26 21595937 PMC3125394

[bib24] Chodniewicz D., and Zhelev D.V. 2003 Chemoattractant receptor-stimulated F-actin polymerization in the human neutrophil is signaled by 2 distinct pathways. Blood. 101:1181–1184. 10.1182/blood-2002-05-1435 12393389

[bib25] Chung C.Y., Lee S., Briscoe C., Ellsworth C., and Firtel R.A. 2000 Role of Rac in controlling the actin cytoskeleton and chemotaxis in motile cells. Proc. Natl. Acad. Sci. USA. 97:5225–5230. 10.1073/pnas.97.10.5225 10805781 PMC25810

[bib26] Collins S.J., Gallo R.C., and Gallagher R.E. 1977 Continuous growth and differentiation of human myeloid leukaemic cells in suspension culture. Nature. 270:347–349. 10.1038/270347a0 271272

[bib27] Collins S.J., Ruscetti F.W., Gallagher R.E., and Gallo R.C. 1978 Terminal differentiation of human promyelocytic leukemia cells induced by dimethyl sulfoxide and other polar compounds. Proc. Natl. Acad. Sci. USA. 75:2458–2462. 10.1073/pnas.75.5.2458 276884 PMC392573

[bib28] Dayel M.J., and King N. 2014 Prey capture and phagocytosis in the choanoflagellate *Salpingoeca rosetta*. PLoS One. 9:e95577 10.1371/journal.pone.0095577 24806026 PMC4012994

[bib29] Deacon J.W., and Saxena G. 1997 Orientated zoospore attachment and cyst germination in *Catenaria anguillulae*, a facultative endoparasite of nematodes. Mycol. Res. 101:513–522. 10.1017/S0953756296003085

[bib30] Demaison C., Parsley K., Brouns G., Scherr M., Battmer K., Kinnon C., Grez M., and Thrasher A.J. 2002 High-level transduction and gene expression in hematopoietic repopulating cells using a human immunodeficiency virus type 1-based lentiviral vector containing an internal spleen focus forming virus promoter. Hum. Gene Ther. 13:803–813. 10.1089/10430340252898984 11975847

[bib31] Diz-Muñoz A., Thurley K., Chintamen S., Altschuler S.J., Wu L.F., Fletcher D.A., and Weiner O.D. 2016 Membrane tension acts through PLD2 and mTORC2 to limit actin network assembly during neutrophil migration. PLoS Biol. 14:e1002474 10.1371/journal.pbio.1002474 27280401 PMC4900667

[bib32] Dovas A., Gevrey J.-C., Grossi A., Park H., Abou-Kheir W., and Cox D. 2009 Regulation of podosome dynamics by WASp phosphorylation: implication in matrix degradation and chemotaxis in macrophages. J. Cell Sci. 122:3873–3882. 10.1242/jcs.051755 19808890 PMC2773189

[bib33] Edelstein A., Amodaj N., Hoover K., Vale R., and Stuurman N. 2010 Computer control of microscopes using µManager. Curr. Protoc. Mol. Biol. 14:20 10.1002/0471142727.mb1420s92 20890901 PMC3065365

[bib34] Fritz-Laylin L.K., Prochnik S.E., Ginger M.L., Dacks J.B., Carpenter M.L., Field M.C., Kuo A., Paredez A., Chapman J., Pham J., 2010 The genome of *Naegleria gruberi* illuminates early eukaryotic versatility. Cell. 140:631–642. 10.1016/j.cell.2010.01.032 20211133

[bib35] Fulton C. 1970 Amebo-flagellates as research partners: The laboratory biology of *Naegleria* and *Tetramitus*. *In* Methods Cell Physiol. Prescott D.M., editor. Academic Press, New York. 341–476.

[bib36] Gautier J.J., Lomakina M.E., Bouslama-Oueghlani L., Derivery E., Beilinson H., Faigle W., Loew D., Louvard D., Echard A., Alexandrova A.Y., 2011 Clathrin is required for Scar/Wave-mediated lamellipodium formation. J. Cell Sci. 124:3414–3427. 10.1242/jcs.081083 22010197

[bib37] Gleason F.H., and Lilje O. 2009 Structure and function of fungal zoospores: ecological implications. Fungal Ecol. 2:53–59. 10.1016/j.funeco.2008.12.002

[bib38] Goodson H.V., and Hawse W.F. 2002 Molecular evolution of the actin family. J. Cell Sci. 115:2619–2622. 12077353 10.1242/jcs.115.13.2619

[bib39] Guerriero C.J., and Weisz O.A. 2007 N-WASP inhibitor wiskostatin nonselectively perturbs membrane transport by decreasing cellular ATP levels. Am. J. Physiol. Cell Physiol. 292:C1562–C1566. 10.1152/ajpcell.00426.2006 17092993

[bib40] Heinrich V., and Lee C.-Y. 2011 Blurred line between chemotactic chase and phagocytic consumption: an immunophysical single-cell perspective. J. Cell Sci. 124:3041–3051. 10.1242/jcs.086413 21914817 PMC3172184

[bib41] Hertel L.A., Bayne C.J., and Loker E.S. 2002 The symbiont *Capsaspora owczarzaki*, nov. gen. nov. sp., isolated from three strains of the pulmonate snail *Biomphalaria glabrata* is related to members of the Mesomycetozoea. Int. J. Parasitol. 32:1183–1191. 10.1016/S0020-7519(02)00066-8 12117501

[bib42] Ishihara D., Dovas A., Park H., Isaac B.M., and Cox D. 2012 The chemotactic defect in Wiskott-Aldrich syndrome macrophages is due to the reduced persistence of directional protrusions. PLoS One. 7:e30033 10.1371/journal.pone.0030033 22279563 PMC3261183

[bib43] Jain N., and Thanabalu T. 2015 Molecular difference between WASP and N-WASP critical for chemotaxis of T-cells towards SDF-1α. Sci. Rep. 5:15031 10.1038/srep15031 26463123 PMC4604493

[bib44] James T.Y., Kauff F., Schoch C.L., Matheny P.B., Hofstetter V., Cox C.J., Celio G., Gueidan C., Fraker E., Miadlikowska J., 2006 Reconstructing the early evolution of Fungi using a six-gene phylogeny. Nature. 443:818–822. 10.1038/nature05110 17051209

[bib45] Jones G.E., Zicha D., Dunn G.A., Blundell M., and Thrasher A. 2002 Restoration of podosomes and chemotaxis in Wiskott-Aldrich syndrome macrophages following induced expression of WASp. Int. J. Biochem. Cell Biol. 34:806–815. 10.1016/S1357-2725(01)00162-5 11950596

[bib46] Jones R.A., Feng Y., Worth A.J., Thrasher A.J., Burns S.O., and Martin P. 2013 Modelling of human Wiskott-Aldrich syndrome protein mutants in zebrafish larvae using in vivo live imaging. J. Cell Sci. 126:4077–4084. 10.1242/jcs.128728 23868979 PMC3772384

[bib47] Kim J.H., Lee S.-R., Li L.-H., Park H.-J., Park J.-H., Lee K.Y., Kim M.-K., Shin B.A., and Choi S.-Y. 2011 High cleavage efficiency of a 2A peptide derived from porcine teschovirus-1 in human cell lines, zebrafish and mice. PLoS One. 6:e18556 10.1371/journal.pone.0018556 21602908 PMC3084703

[bib48] Knoll A.H. 2014 Paleobiological perspectives on early eukaryotic evolution. Cold Spring Harb. Perspect. Biol. 6:a016121 10.1101/cshperspect.a016121 24384569 PMC3941219

[bib49] Koch W.J. 1968 Studies of the motile cells of chytrids. V. Flagellar retraction in posteriorly uniflagellate fungi. Am. J. Bot. 55:841 10.2307/2440973

[bib50] Kollmar M., Lbik D., and Enge S. 2012 Evolution of the eukaryotic ARP2/3 activators of the WASP family: WASP, WAVE, WASH, and WHAMM, and the proposed new family members WAWH and WAML. BMC Res. Notes. 5:88 10.1186/1756-0500-5-88 22316129 PMC3298513

[bib51] Koronakis V., Hume P.J., Humphreys D., Liu T., Hørning O., Jensen O.N., and McGhie E.J. 2011 WAVE regulatory complex activation by cooperating GTPases Arf and Rac1. Proc. Natl. Acad. Sci. USA. 108:14449–14454. 10.1073/pnas.1107666108 21844371 PMC3167530

[bib52] Kumar S., Xu J., Perkins C., Guo F., Snapper S., Finkelman F.D., Zheng Y., and Filippi M.-D. 2012 Cdc42 regulates neutrophil migration via crosstalk between WASp, CD11b, and microtubules. Blood. 120:3563–3574. 10.1182/blood-2012-04-426981 22932798 PMC3482864

[bib53] Kusdian G., Woehle C., Martin W.F., and Gould S.B. 2013 The actin-based machinery of *Trichomonas vaginalis* mediates flagellate-amoeboid transition and migration across host tissue. Cell. Microbiol. 15:1707–1721. 23530917 10.1111/cmi.12144

[bib54] Lämmermann T., and Sixt M. 2009 Mechanical modes of ‘amoeboid’ cell migration. Curr. Opin. Cell Biol. 21:636–644. 10.1016/j.ceb.2009.05.003 19523798

[bib55] Lämmermann T., Bader B.L., Monkley S.J., Worbs T., Wedlich-Söldner R., Hirsch K., Keller M., Förster R., Critchley D.R., Fässler R., and Sixt M. 2008 Rapid leukocyte migration by integrin-independent flowing and squeezing. Nature. 453:51–55. 10.1038/nature06887 18451854

[bib56] Liu Y.-J., Le Berre M., Lautenschlaeger F., Maiuri P., Callan-Jones A., Heuzé M., Takaki T., Voituriez R., and Piel M. 2015 Confinement and low adhesion induce fast amoeboid migration of slow mesenchymal cells. Cell. 160:659–672. 10.1016/j.cell.2015.01.007 25679760

[bib57] Lommel S., Benesch S., Rottner K., Franz T., Wehland J., and Kühn R. 2001 Actin pedestal formation by enteropathogenic *Escherichia coli* and intracellular motility of *Shigella flexneri* are abolished in N-WASP-defective cells. EMBO Rep. 2:850–857. 10.1093/embo-reports/kve197 11559594 PMC1084051

[bib58] Longcore J.E., Pessier A.P., and Nichols D.K. 1999 *Batrachochytrium Dendrobatidis* gen. et sp. nov., a chytrid pathogenic to amphibians. Mycologia. 91:219–227. 10.2307/3761366

[bib59] Maugis B., Brugués J., Nassoy P., Guillen N., Sens P., and Amblard F. 2010 Dynamic instability of the intracellular pressure drives bleb-based motility. J. Cell Sci. 123:3884–3892. 10.1242/jcs.065672 20980385

[bib60] McGraw T.E., and Subtil A. 2001 Endocytosis: Biochemical analyses. Curr. Protoc. Cell Biol. 15:3 10.1002/0471143030.cb1503s03 18228330

[bib61] Merrifield C.J., Qualmann B., Kessels M.M., and Almers W. 2004 Neural Wiskott Aldrich Syndrome Protein (N-WASP) and the Arp2/3 complex are recruited to sites of clathrin-mediated endocytosis in cultured fibroblasts. Eur. J. Cell Biol. 83:13–18. 10.1078/0171-9335-00356 15085951

[bib62] Miki H., Suetsugu S., and Takenawa T. 1998 WAVE, a novel WASP-family protein involved in actin reorganization induced by Rac. EMBO J. 17:6932–6941. 10.1093/emboj/17.23.6932 9843499 PMC1171041

[bib63] Millius A., and Weiner O.D. 2010 Manipulation of neutrophil-like HL-60 cells for the study of directed cell migration. Methods Mol. Biol. 591:147–158. 10.1007/978-1-60761-404-3_9 19957129 PMC3128798

[bib64] Misra A., Lim R.P.Z., Wu Z., and Thanabalu T. 2007 N-WASP plays a critical role in fibroblast adhesion and spreading. Biochem. Biophys. Res. Commun. 364:908–912. 10.1016/j.bbrc.2007.10.086 17963692

[bib65] Moreau V., Frischknecht F., Reckmann I., Vincentelli R., Rabut G., Stewart D., and Way M. 2000 A complex of N-WASP and WIP integrates signalling cascades that lead to actin polymerization. Nat. Cell Biol. 2:441–448. 10.1038/35017080 10878810

[bib66] Myers S.A., Han J.W., Lee Y., Firtel R.A., and Chung C.Y. 2005 A *Dictyostelium* homologue of WASP is required for polarized F-actin assembly during chemotaxis. Mol. Biol. Cell. 16:2191–2206. 10.1091/mbc.E04-09-0844 15728724 PMC1087228

[bib67] Naqvi S.N., Zahn R., Mitchell D.A., Stevenson B.J., and Munn A.L. 1998 The WASp homologue Las17p functions with the WIP homologue End5p/verprolin and is essential for endocytosis in yeast. Curr. Biol. 8:959–962. 10.1016/S0960-9822(98)70396-3 9742397

[bib68] Nolen B.J., Tomasevic N., Russell A., Pierce D.W., Jia Z., McCormick C.D., Hartman J., Sakowicz R., and Pollard T.D. 2009 Characterization of two classes of small molecule inhibitors of Arp2/3 complex. Nature. 460:1031–1034. 10.1038/nature08231 19648907 PMC2780427

[bib69] Paluch E.K., and Raz E. 2013 The role and regulation of blebs in cell migration. Curr. Opin. Cell Biol. 25:582–590. 10.1016/j.ceb.2013.05.005 23786923 PMC3989058

[bib70] Paredez A.R., Assaf Z.J., Sept D., Timofejeva L., Dawson S.C., Wang C.-J.R., and Cande W.Z. 2011 An actin cytoskeleton with evolutionarily conserved functions in the absence of canonical actin-binding proteins. Proc. Natl. Acad. Sci. USA. 108:6151–6156. 10.1073/pnas.1018593108 21444821 PMC3076823

[bib71] Parfrey L.W., Lahr D.J.G., Knoll A.H., and Katz L.A. 2011 Estimating the timing of early eukaryotic diversification with multigene molecular clocks. Proc. Natl. Acad. Sci. USA. 108:13624–13629. 10.1073/pnas.1110633108 21810989 PMC3158185

[bib72] Petrie R.J., and Yamada K.M. 2015 Fibroblasts lead the way: A unified view of 3D cell motility. Trends Cell Biol. 25:666–674. 10.1016/j.tcb.2015.07.013 26437597 PMC4628848

[bib73] Pettitt M.E., Orme B.A.A., Blake J.R., and Leadbeater B.S.C. 2002 The hydrodynamics of filter feeding in choanoflagellates. Eur. J. Protistol. 38:313–332. 10.1078/0932-4739-00854

[bib74] Ramsey W.S. 1972 Locomotion of human polymorphonuclear leucocytes. Exp. Cell Res. 72:489–501. 10.1016/0014-4827(72)90019-5 4556707

[bib75] Raper K.B. 1935 Dictyostelium discoideum, a new species of slime mold from decaying forest leaves. J. Agric. Res. 50:135–147.

[bib76] Rodriguez M.A., LeClaire L.L. III, and Roberts T.M. 2005 Preparing to move: Assembly of the MSP amoeboid motility apparatus during spermiogenesis in *Ascaris*. Cell Motil. Cytoskeleton. 60:191–199. 10.1002/cm.20058 15751099

[bib77] Rohatgi R., Ma L., Miki H., Lopez M., Kirchhausen T., Takenawa T., and Kirschner M.W. 1999 The interaction between N-WASP and the Arp2/3 complex links Cdc42-dependent signals to actin assembly. Cell. 97:221–231. 10.1016/S0092-8674(00)80732-1 10219243

[bib78] Rottner K., Hänisch J., and Campellone K.G. 2010 WASH, WHAMM and JMY: regulation of Arp2/3 complex and beyond. Trends Cell Biol. 20:650–661. 10.1016/j.tcb.2010.08.014 20888769

[bib79] Ruprecht V., Wieser S., Callan-Jones A., Smutny M., Morita H., Sako K., Barone V., Ritsch-Marte M., Sixt M., Voituriez R., and Heisenberg C.-P. 2015 Cortical contractility triggers a stochastic switch to fast amoeboid cell motility. Cell. 160:673–685. 10.1016/j.cell.2015.01.008 25679761 PMC4328143

[bib80] Sarmiento C., Wang W., Dovas A., Yamaguchi H., Sidani M., El-Sibai M., Desmarais V., Holman H.A., Kitchen S., Backer J.M., 2008 WASP family members and formin proteins coordinate regulation of cell protrusions in carcinoma cells. J. Cell Biol. 180:1245–1260. 10.1083/jcb.200708123 18362183 PMC2290849

[bib81] Schiefermeier N., Teis D., and Huber L.A. 2011 Endosomal signaling and cell migration. Curr. Opin. Cell Biol. 23:615–620. 10.1016/j.ceb.2011.04.001 21546233 PMC3188704

[bib82] Schindelin J., Arganda-Carreras I., Frise E., Kaynig V., Longair M., Pietzsch T., Preibisch S., Rueden C., Saalfeld S., Schmid B., 2012 Fiji: an open-source platform for biological-image analysis. Nat. Methods. 9:676–682. 10.1038/nmeth.2019 22743772 PMC3855844

[bib83] Sens K.L., Zhang S., Jin P., Duan R., Zhang G., Luo F., Parachini L., and Chen E.H. 2010 An invasive podosome-like structure promotes fusion pore formation during myoblast fusion. J. Cell Biol. 191:1013–1027. 10.1083/jcb.201006006 21098115 PMC2995175

[bib84] Shaner N.C., Lin M.Z., McKeown M.R., Steinbach P.A., Hazelwood K.L., Davidson M.W., and Tsien R.Y. 2008 Improving the photostability of bright monomeric orange and red fluorescent proteins. Nat. Methods. 5:545–551. 10.1038/nmeth.1209 18454154 PMC2853173

[bib85] Shi Y., Dong B., Miliotis H., Liu J., Alberts A.S., Zhang J., and Siminovitch K.A. 2009 Src kinase Hck association with the WASp and mDia1 cytoskeletal regulators promotes chemoattractant-induced Hck membrane targeting and activation in neutrophils. Biochem. Cell Biol. 87:207–216. 10.1139/O08-130 19234535

[bib86] Small J.V., and Rottner K. 2010 Elementary Cellular Processes Driven by Actin Assembly: Lamellipodia and Filopodia. *In* Actin-based Motility. Springer Netherlands, Dordrecht, Netherlands. 3–33.

[bib87] Snapper S.B., Takeshima F., Antón I., Liu C.H., Thomas S.M., Nguyen D., Dudley D., Fraser H., Purich D., Lopez-Ilasaca M., 2001 N-WASP deficiency reveals distinct pathways for cell surface projections and microbial actin-based motility. Nat. Cell Biol. 3:897–904. 10.1038/ncb1001-897 11584271

[bib88] Snapper S.B., Meelu P., Nguyen D., Stockton B.M., Bozza P., Alt F.W., Rosen F.S., von Andrian U.H., and Klein C. 2005 WASP deficiency leads to global defects of directed leukocyte migration in vitro and in vivo. J. Leukoc. Biol. 77:993–998. 10.1189/jlb.0804444 15774550

[bib89] Srinivasan S., Wang F., Glavas S., Ott A., Hofmann F., Aktories K., Kalman D., and Bourne H.R. 2003 Rac and Cdc42 play distinct roles in regulating PI(3,4,5)P_3_ and polarity during neutrophil chemotaxis. J. Cell Biol. 160:375–385. 10.1083/jcb.200208179 12551955 PMC2172671

[bib90] Stajich J.E., Berbee M.L., Blackwell M., Hibbett D.S., James T.Y., Spatafora J.W., and Taylor J.W. 2009 The fungi. Curr. Biol. 19:R840–R845. 10.1016/j.cub.2009.07.004 19788875 PMC2913116

[bib91] Steffen A., Rottner K., Ehinger J., Innocenti M., Scita G., Wehland J., and Stradal T.E.B. 2004 Sra-1 and Nap1 link Rac to actin assembly driving lamellipodia formation. EMBO J. 23:749–759. 10.1038/sj.emboj.7600084 14765121 PMC380996

[bib92] Stradal T.E.B., and Scita G. 2006 Protein complexes regulating Arp2/3-mediated actin assembly. Curr. Opin. Cell Biol. 18:4–10. 10.1016/j.ceb.2005.12.003 16343889

[bib93] Tilney L.G., Hatano S., Ishikawa H., and Mooseker M.S. 1973 The polymerization of actin: Its role in the generation of the acrosomal process of certain echinoderm sperm. J. Cell Biol. 59:109–126. 10.1083/jcb.59.1.109 4356568 PMC2110911

[bib94] Traynor D., and Kay R.R. 2007 Possible roles of the endocytic cycle in cell motility. J. Cell Sci. 120:2318–2327. 10.1242/jcs.007732 17606987

[bib95] Veltman D.M., and Insall R.H. 2010 WASP family proteins: Their evolution and its physiological implications. Mol. Biol. Cell. 21:2880–2893. 10.1091/mbc.E10-04-0372 20573979 PMC2921111

[bib96] Veltman D.M., King J.S., Machesky L.M., and Insall R.H. 2012 SCAR knockouts in *Dictyostelium*: WASP assumes SCAR’s position and upstream regulators in pseudopods. J. Cell Biol. 198:501–508. 10.1083/jcb.201205058 22891261 PMC3514037

[bib97] Weiner O.D., Rentel M.C., Ott A., Brown G.E., Jedrychowski M., Yaffe M.B., Gygi S.P., Cantley L.C., Bourne H.R., and Kirschner M.W. 2006 Hem-1 complexes are essential for Rac activation, actin polymerization, and myosin regulation during neutrophil chemotaxis. PLoS Biol. 4:e38 10.1371/journal.pbio.0040038 16417406 PMC1334198

[bib98] Weiner O.D., Marganski W.A., Wu L.F., Altschuler S.J., and Kirschner M.W. 2007 An actin-based wave generator organizes cell motility. PLoS Biol. 5:e221 10.1371/journal.pbio.0050221 17696648 PMC1945041

[bib99] Winter D., Podtelejnikov A.V., Mann M., and Li R. 1997 The complex containing actin-related proteins Arp2 and Arp3 is required for the motility and integrity of yeast actin patches. Curr. Biol. 7:519–529. 10.1016/S0960-9822(06)00223-5 9210376

[bib100] Worth A.J.J., Metelo J., Bouma G., Moulding D., Fritzsche M., Vernay B., Charras G., Cory G.O.C., Thrasher A.J., and Burns S.O. 2013 Disease-associated missense mutations in the EVH1 domain disrupt intrinsic WASp function causing dysregulated actin dynamics and impaired dendritic cell migration. Blood. 121:72–84. 10.1182/blood-2012-01-403857 23160469 PMC3779380

[bib101] Yoshida K., and Soldati T. 2006 Dissection of amoeboid movement into two mechanically distinct modes. J. Cell Sci. 119:3833–3844. 10.1242/jcs.03152 16926192

[bib102] Zalevsky J., Lempert L., Kranitz H., and Mullins R.D. 2001 Different WASP family proteins stimulate different Arp2/3 complex-dependent actin-nucleating activities. Curr. Biol. 11:1903–1913. 10.1016/S0960-9822(01)00603-0 11747816

[bib103] Zhang H., Schaff U.Y., Green C.E., Chen H., Sarantos M.R., Hu Y., Wara D., Simon S.I., and Lowell C.A. 2006 Impaired integrin-dependent function in Wiskott-Aldrich syndrome protein-deficient murine and human neutrophils. Immunity. 25:285–295. 10.1016/j.immuni.2006.06.014 16901726 PMC4698343

[bib104] Zhelev D.V., Alteraifi A.M., and Chodniewicz D. 2004 Controlled pseudopod extension of human neutrophils stimulated with different chemoattractants. Biophys. J. 87:688–695. 10.1529/biophysj.103.036699 15240502 PMC1304392

[bib105] Zhu Z., Chai Y., Jiang Y., Li W., Hu H., Li W., Wu J.-W., Wang Z.-X., Huang S., and Ou G. 2016 Functional coordination of WAVE and WASP in *C. elegans* neuroblast migration. Dev. Cell. 39:224–238. 10.1016/j.devcel.2016.09.029 27780040

[bib106] Zicha D., Allen W.E., Brickell P.M., Kinnon C., Dunn G.A., Jones G.E., and Thrasher A.J. 1998 Chemotaxis of macrophages is abolished in the Wiskott-Aldrich syndrome. Br. J. Haematol. 101:659–665. 10.1046/j.1365-2141.1998.00767.x 9674738

