# Supplementary material for: WASP and SCAR are evolutionarily conserved in actin-filled pseudopod-based motility
Source: J Cell Biol. 2017 Jun 5;216(6):1673–88. doi: 10.1083/jcb.201701074 (PMC5461030; doi:10.1083/jcb.201701074)
Supplement: Supplemental Materials (PDF) [file JCB_201701074_sm.pdf]

Fritz-Laylin et al., https://doi.org/10.1083/jcb.201701074

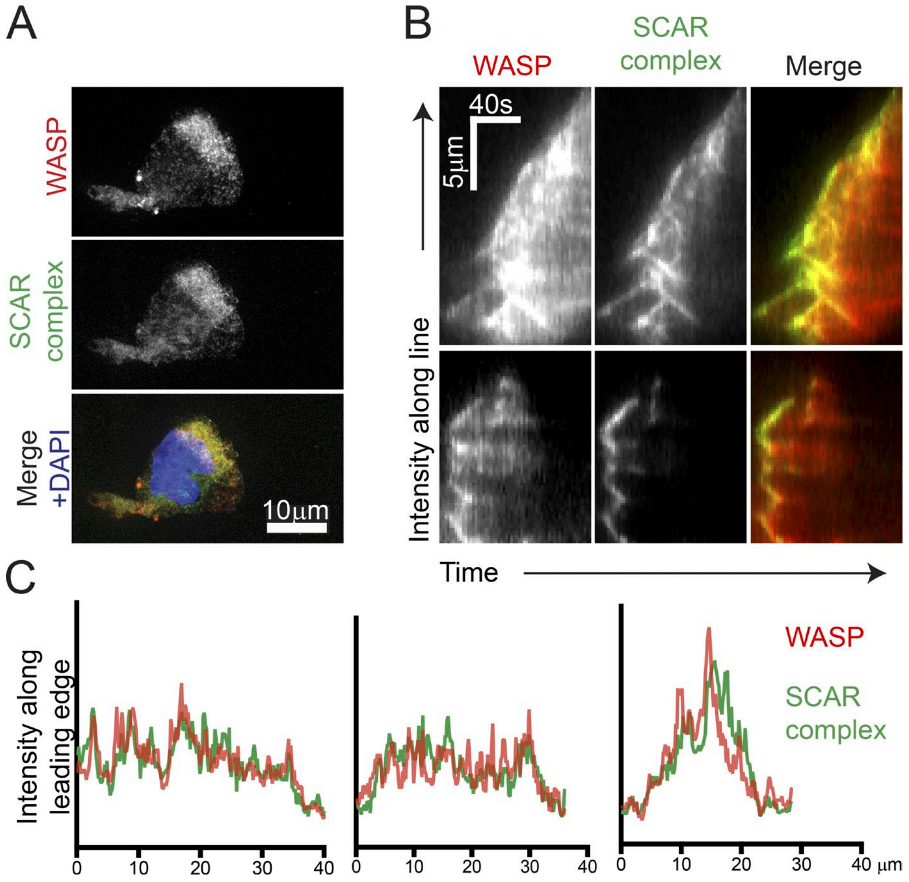

Figure S1. **WASP localizes to pseudopods of migrating neutrophils.** (A) Maximum-projection image of localization of TagRFP-WASP and Hem-1–YFP (a member of the SCAR regulatory complex) expressed in differentiated HL-60 cells fixed while migrating on a fibronectin-coated surface. (B) Kymographs of pseudopods of two live HL-60 cells showing colocalization over time of TagRFP-WASP and Hem-1–YFP patterns. Time is from left (T = 0) to right, and direction is from bottom (inside cell) to top (outside cell). See Video 1 for the cells from which these kymographs were generated. (C) Line scans following the contour of the leading edge of each individual cell shown Fig. 1 (A and B) showing that TagRFP-WASP (red) partially colocalizes with Hem-1–YFP (green).

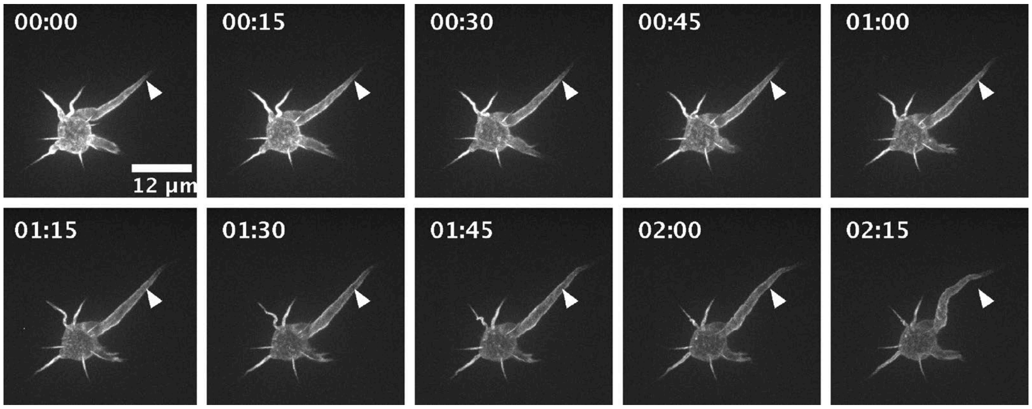

Figure S2. **WASP depletion in neutrophils leads to dynamic rhino protrusions.** Time-lapse microscopy of a live WASP-KD HL-60 cell with aberrant rhino protrusions, with polymerized actin visualized using Utrophin261-mCherry. Time is shown in min:s, and an immobile arrowhead highlights protrusion dynamics. The 01:15 time point of this cell is shown in Fig. 2 F on the right.

THE JOURNAL OF CELL BIOLOGY

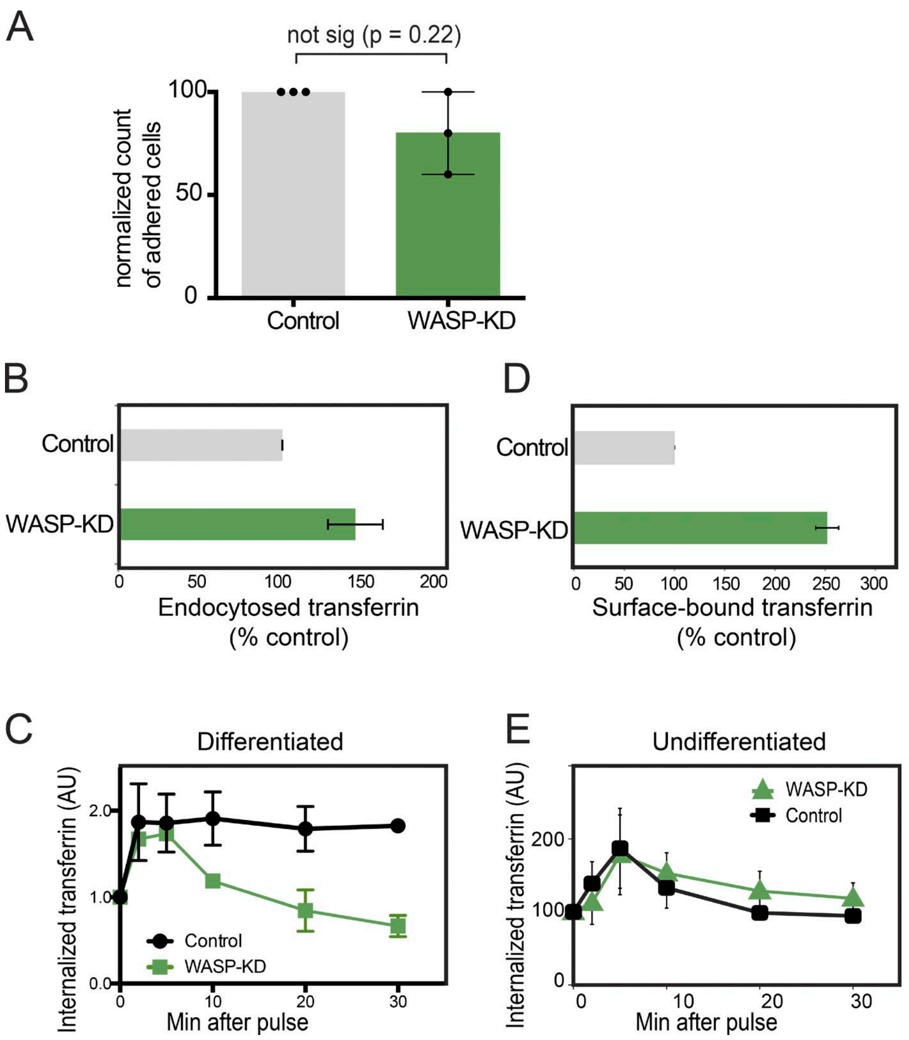

Figure S3. **WASP is not required for adhesion or endocytosis by HL-60 cells.** (A) WASP-KD does not significantly reduce HL-60 cells' ability to adhere to fibronectin-coated surfaces. Bars represent means from three biological replicates normalized to an internal control in each experiment. The p-value was obtained from a two-tailed paired *t* test. At least 1,000 cells were counted for each internal control. (B) Steady-state endocytosis was measured for WASP-KD (green) and control cells (gray) by incubating cells for 10 min at 37°C with fluorescent transferrin and then immediately washing with ice-cold acid buffer to remove surface-bound transferrin. Cells were then fixed, and endocytosed transferrin was quantified by FACS analysis. (C) Quantification of actin-mediated endocytosis and receptor recycling in differentiated (neutrophil-like) control (black circles) and WASP-KD HL-60 cells (green squares). Cells were incubated with fluorescent transferrin, placed at 37°C for the indicated time, washed with ice-cold acid buffer to remove surface-bound transferrin, and fixed for FACS analysis. (D) Transferrin receptor density was measured by incubating cells at 37°C in serum-free medium (to remove surface-bound transferrin), chilling cells, and then incubating on ice with fluorescent transferrin. Cells were then washed with PBS to remove unbound transferrin and fixed. Surface-bound transferrin was then quantified by FACS analysis. (B and D) Values were normalized to the percent control within each of three independent experiments, with 10,000 cells analyzed for each sample. (E) Quantification of actin-mediated endocytosis and receptor recycling in undifferentiated control (black squares) and WASP-KD HL-60 cells (green triangles). Cells were prepared as in C. Note that the recycling assays are normalized. For comparing absolute amounts of material endocytosed, refer to B. Within each experiment, samples for each cell line were normalized to time 0. Means and SD for three independent experiments are shown, with 10,000 cells analyzed for each sample. AU, arbitrary unit.

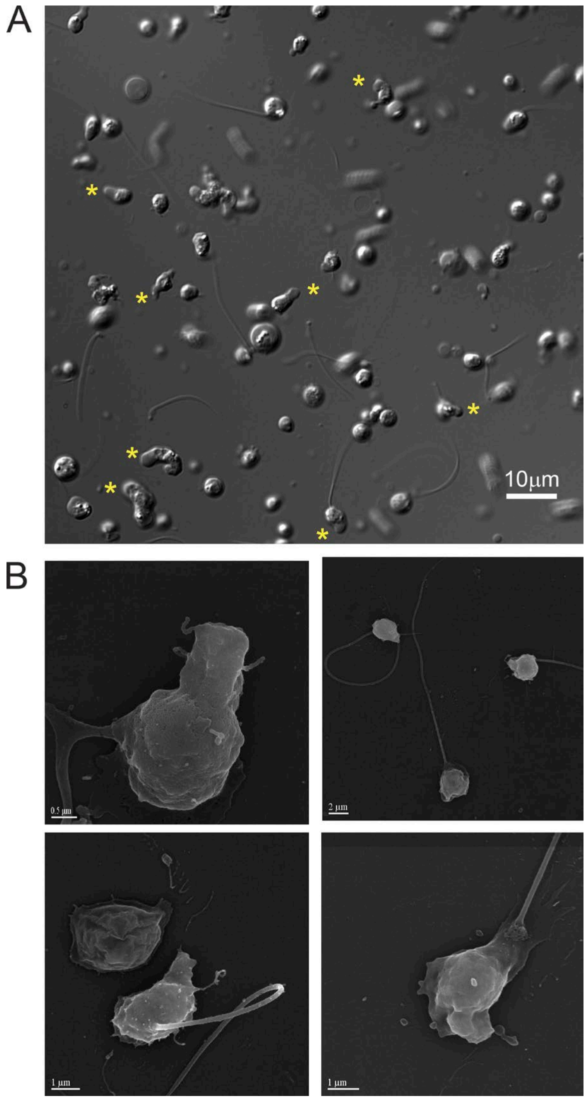

Figure S4. **Additional examples of chytrid pseudopods.** (A) Differential interference contrast image of a representative field of synchronized Bd zoospores. Asterisks highlight cells with obvious pseudopods. Rapidly swimming flagellate cells are blurred. (B) Additional examples of scanning electron micrographs of chytrid zoospores.

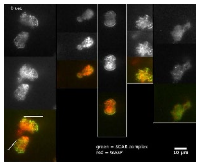

Video 1. **Five examples of time-lapse videos showing TIRF microscopy of live differentiated HL-60 neutrophil cells expressing Hem-1–YFP and TagRFP-WASP.** Hem-1–YFP is on the top and is green in the overlay; TagRFP-WASP is in the middle and is red in the overlay. White lines indicate positions for kymographs in Fig. S1 B. One frame was acquired every 2 s, and video is displayed at 10 frames per second.

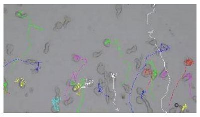

Video 2. **Migration of control differentiated HL-60 neutrophil cells and of differentiated WASP-KD HL-60 cells in a 2D environment (EZ-TAXIScan assay).** Cells expressing control shRNA migrating in a chemoattractant gradient (source is at top) between two glass surfaces with 5-μm spacing. All cells initially within the field of view were manually tracked as shown. Many WASP-KD cells exhibited the rhino phenotype, and their motility was effectively abolished (e.g., cells 11, 32, 33, 39, and 42). One frame was acquired every 20 s, and video is displayed at 15 frames per second.

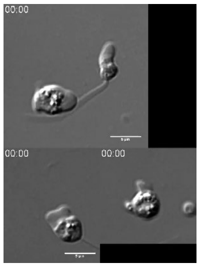

Video 3. **Time-lapse videos showing the representative examples of Bd chytrid zoospores with pseudopods imaged using differential interference contrast microscopy pictured in Fig. 4 B, including a cell with flagella (bottom left), a cell without a flagellum (bottom right), and one cell of each (top).** One frame was acquired per second, and video is displayed at four frames per second.

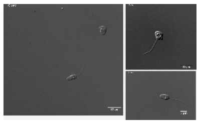

Video 4. **Examples of Bd chytrid cells crawling between two glass coverslips separated by 1-μm glass beads.** See also Fig. 5. One frame was acquired per second, and video is displayed at 20 frames per second.

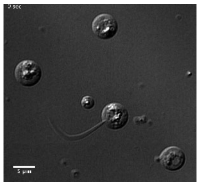

Video 5. **Time-lapse imaging showing an example of flagellar retraction by Bd chytrid zoospores.** One frame was acquired every 0.1 s, and video is displayed at 20 frames per second.

Table S1. **Fast/blood cell (expressing WASP)**

References (PMID)	Cell type or types	Finding
Jones et al., 2002 (11950596)	Macrophages (from blood of human Wiskott-Aldrich syndrome patients)	Migrating macrophages that lack WASP fail to form actin-rich protrusions. WASP-deficient cells moved aberrantly and did not have directionality toward a chemoattractant. See Fig. 2.
Burns et al., 2001 (11493463)	Dendritic cells (from blood of human Wiskott-Aldrich syndrome patients)	Expressing WASP restores actin- and WASP-filled protrusions. Arp2/3 is also shown to be enriched in the protrusions. "Persistent broad, leading-edge lamellipodia do not form" and translocation is "severely compromised" in WASP-deficient cells. See Fig. 5.
Badolato et al., 1998 (9670984)	Monocytes (from human Wiskott-Aldrich syndrome patients)	Cells from Wiskott-Aldrich syndrome patients remained rounded upon stimulation with chemoattractant, and their migration was severely impaired; normal monocytes in their assay showed a polarized actin distribution and readily formed pseudopods.
Binks et al., 1998 (9808195)	Dendritic cells (from human Wiskott-Aldrich syndrome patients)	Cells from Wiskott-Aldrich syndrome patients were "unable to polarize normally and have severely reduced translocational motility in vitro." (Although they did still see "ruffles" in the WASP-deficient cells, they did not observe larger lamellar structures.)
Zicha et al., 1998 (9674738)	Macrophages and neutrophils (from human Wiskott-Aldrich syndrome patients)	Macrophages from Wiskott-Aldrich syndrome patients had defects in directional chemotaxis, although neutrophils from the same patients were not disrupted. Both mutant and wild-type cells moved at approximately the same speed. Notably, the statistical analysis in this paper eliminated any cell that did not reach a certain distance (60 μm for neutrophils and 10 μm for macrophages), and therefore ignored cells with severely impaired motility.
Linder et al., 1999 (10449748)	Macrophages (from human Wiskott-Aldrich syndrome patients)	Cells from Wiskott-Aldrich syndrome patients formed fewer podosomes and filopodia, which were distributed around the entire cell instead of just at the leading edge.
Snapper et al., 2005 (15774550)	Neutrophils (WASP-KO mouse)	Defect in chemotaxis in WASP-deficient cells (by 25–50%).
Kumar et al., 2012 (22932798)	Neutrophils (WASP-KO mouse)	Cdc42 controls neutrophil chemotaxis and polarity via WASP. "WASP$^{-/-}$ neutrophils have defective chemotaxis and exhibit loss of polarity." Cells lacking WASP exhibit significantly lower speed ("Sp") and straightness ("St."). The authors also report that WASP-deficient cells exhibit a huge increase in the number of protrusions, with many smaller protrusions occurring on the sides of cells instead of at the front.
Anderson et al., 2003 (12529859)	Neutrophils (blood from healthy humans)	Neutrophils were loaded with purified SCAR or WASP peptides. High concentrations of SCAR severely disrupted motility, and WASP had a smaller effect.
Zhang et al., 2006 (16901726)	Neutrophils (WASP-KO mouse)	WASP-deficient cells had impaired adhesion and remain unpolarized, rounded, and without protrusions. Their transendothelial migration was also severely disrupted.
Jones et al., 2013 (23868979)	Neutrophils (zebrafish)	Inside zebrafish embryos, reduced protrusions and cell velocity in cells with UAS-WASP mutant. See Fig. 1 G.
Shi et al., 2009 (19234535)	Neutrophils (WASP-KO mouse)	WASP localizes to pseudopods of chemotacting cells. See Fig. 1 C.
Dovas et al., 2009 (19808890)	Macrophages (mouse and human)	Phosphorylation/dephosphorylation of WASP was required for normal podosome formation and turnover as well as fibronectin matrix degradation. Chemotaxis was impaired by RNAi-reduction of WASP and was rescued by adding WASP, but not by a phosphorylation mutant.
Ishihara et al., 2012 (22279563)	Macrophages (WASP-KO mouse)	WASP is responsible for an initial wave of actin polymerization in response to global stimulation with chemoattractant. Protrusions from WASP-deficient cells were directional, showing intact directional sensing. However, the protrusions from WASP-deficient cells demonstrated reduced persistence compared to wild-type cells.
Myers et al., 2005 (15728724)	Dictyostelium	Cells with reduced levels of WASP exhibit defects in polarized actin assembly, cell migration, and chemotaxis.
Veltman et al., 2012 (22891261)	Dictyostelium	When SCAR/WAVE is knocked out, WASP assumes the localization and presumably some of the functions of SCAR/WAVE.
Jain and Thanabalu, 2015 (26463123)	Jurkat T cells (human)	Knocking down WASP in Jurkat T cells slowed motility and abolished directionality. Overexpression of N-WASP in WASP-KD cells restored the migration velocity without correcting the chemotactic defect. However, insertion of a section of the WASP amino acid sequence into N-WASP enabled N-WASP to rescue the chemotactic defect of WASP-KD cells.
Worth et al., 2013 (23160469)	Dendritic (WASP-KO mouse)	Cells lacking WASP form multiple unpolarized lamellipodia and exhibit migration defects (in persistence and directionality, although not speed). Expressing exogenous WASP rescues normal protrusions and migration.
Blundell et al., 2008 (18388921)	Dendritic (WASP-KO mouse)	Number of podosome protrusions as reduced in WASP-KD cells and could be rescued by transducing with WASP. Speed of WASP-KD cells was drastically reduced compared to wild-type and WASP-rescue cells.
Zhu et al., 2016 (27780040)	Neuroblasts (*C. Elegans*)	Migrating neuroblasts in developing worms use both WASP and SCAR/WAVE. SCAR mutations reduced migration and WASP mutation further impaired motility in SCAR-deficient cells.

Table S2. **Cells with adhesion-based motility (expressing N-WASP)**

Reference (PMID)	Cell type or types	Finding
Misra et al., 2007 (17963692)	Adherent fibroblasts (mouse N-WASP$^{del/del}$ cell line)	N-WASP deletion disrupts adhesion.
Bryce et al., 2005 (16051170)	Adherent fibrosarcoma cells (human)	N-WASP KD by siRNA did not reduce lamellipodia formation. By removing an N-WASP activator from cells, the lamellipodia were not as persistent, cell migration was defective, and fewer adhesions formed.
Desmarais et al., 2009 (19373774)	Carcinoma cells (rat)	Cells depleted of N-WASP using siRNA show a defect in in vadopodium-based chemotaxis.
Sarmiento et al., 2008 (18362183)	Carcinoma cells (rat)	siRNA WAVE depletion inhibited lamellipodia formation to a greater degree than N-WASP. Depleting both resulted in aberrant jagged protrusion.
Benseñor et al., 2007 (17264147)	Slowly crawling MDBK (bovine)	N-WASP, Arp2/3, and actin all localize to protrusions. N-WASP is required for FGF2-stimulated migration.
Lommel et al., 2001 (11559594)	Adherent fibroblasts (mouse N-WASP$^{flox/flox}$)	Adherent cells lacking N-WASP still form filopodia.
Tang et al., 2013 (23273897)	Carcinoma cells (human A431 and HeLa cell lines)	"N-WASP has a crucial proinvasive role in driving Arp2/3 complex-mediated actin assembly in cooperation with FAK at invasive cell edges, but WRC depletion can promote 3D cell motility."
Snapper et al., 2001 (11584271)	Fibroblasts (MEFs isolated from N-WASP$^{+/+}$ and N-WASP$^{neo/neo}$)	N-WASP is dispensable for lamellipodia and filopodia formation in fibroblasts.
Mizutani et al., 2002 (11830518)	Fibroblasts (rat)	N-WASP is essential for podosome adhesion structures and degrading extracellular matrix.

References

Anderson, S.I., B. Behrendt, L.M. Machesky, R.H. Insall, and G.B. Nash. 2003. Linked regulation of motility and integrin function in activated migrating neutrophils revealed by interference in remodelling of the cytoskeleton. *Cell Motil. Cytoskeleton.* 54:135–146. http://dx.doi.org/10.1002/cm.10091

Badolato, R., S. Sozzani, F. Malacarne, S. Bresciani, M. Fiorini, A. Borsatti, A. Albertini, A. Mantovani, A.G. Ugazio, and L.D. Notarangelo. 1998. Monocytes from Wiskott-Aldrich patients display reduced chemotaxis and lack of cell polarization in response to monocyte chemoattractant protein-1 and formyl-methionyl-leucyl-phenylalanine. *J. Immunol.* 161:1026–1033.

Benseñor, L.B., H.-M. Kan, N. Wang, H. Wallrabe, L.A. Davidson, Y. Cai, D.A. Schafer, and G.S. Bloom. 2007. IQGAP1 regulates cell motility by linking growth factor signaling to actin assembly. *J. Cell Sci.* 120:658–669. http://dx.doi.org/10.1242/jcs.03376

Binks, M., G.E. Jones, P.M. Brickell, C. Kinnon, D.R. Katz, and A.J. Thrasher. 1998. Intrinsic dendritic cell abnormalities in Wiskott-Aldrich syndrome. *Eur. J. Immunol.* 28:3259–3267.

Blundell, M.P., G. Bouma, Y. Calle, G.E. Jones, C. Kinnon, and A.J. Thrasher. 2008. Improvement of migratory defects in a murine model of Wiskott-Aldrich syndrome gene therapy. *Mol. Ther.* 16:836–844. http://dx.doi.org/10.1038/mt.2008.43

Bryce, N.S., E.S. Clark, J.L. Leysath, J.D. Currie, D.J. Webb, and A.M. Weaver. 2005. Cortactin promotes cell motility by enhancing lamellipodial persistence. *Curr. Biol.* 15:1276–1285. http://dx.doi.org/10.1016/j.cub.2005.06.043

Burns, S., A.J. Thrasher, M.P. Blundell, L. Machesky, and G.E. Jones. 2001. Configuration of human dendritic cell cytoskeleton by Rho GTPases, the WAS protein, and differentiation. *Blood.* 98:1142–1149.

Desmarais, V., H. Yamaguchi, M. Oser, L. Soon, G. Mouneimne, C. Sarmiento, R. Eddy, and J. Condeelis. 2009. N-WASP and cortactin are involved in invadopodium-dependent chemotaxis to EGF in breast tumor cells. *Cell Motil. Cytoskeleton.* 66:303–316. http://dx.doi.org/10.1002/cm.20361

Dovas, A., J.-C. Gevrey, A. Grossi, H. Park, W. Abou-Kheir, and D. Cox. 2009. Regulation of podosome dynamics by WASp phosphorylation: implication in matrix degradation and chemotaxis in macrophages. *J. Cell Sci.* 122:3873–3882. http://dx.doi.org/10.1242/jcs.051755

Ishihara, D., A. Dovas, H. Park, B.M. Isaac, and D. Cox. 2012. The chemotactic defect in Wiskott-Aldrich syndrome macrophages is due to the reduced persistence of directional protrusions. *PLoS One.* 7:e30033. http://dx.doi.org/10.1371/journal.pone.0030033

Jain, N., and T. Thanabalu. 2015. Molecular difference between WASP and N-WASP critical for chemotaxis of T-cells towards SDF-1α. *Sci. Rep.* 5:15031. http://dx.doi.org/10.1038/srep15031

Jones, G.E., D. Zicha, G.A. Dunn, M. Blundell, and A. Thrasher. 2002. Restoration of podosomes and chemotaxis in Wiskott-Aldrich syndrome macrophages following induced expression of WASp. *Int. J. Biochem. Cell Biol.* 34:806–815.

Jones, R.A., Y. Feng, A.J. Worth, A.J. Thrasher, S.O. Burns, and P. Martin. 2013. Modelling of human Wiskott-Aldrich syndrome protein mutants in zebrafish larvae using in vivo live imaging. *J. Cell Sci.* 126:4077–4084. http://dx.doi.org/10.1242/jcs.128728

Kumar, S., J. Xu, C. Perkins, F. Guo, S. Snapper, F.D. Finkelman, Y. Zheng, and M.-D. Filippi. 2012. Cdc42 regulates neutrophil migration via crosstalk between WASp, CD11b, and microtubules. *Blood.* 120:3563–3574. http://dx.doi.org/10.1182/blood-2012-04-426981

Linder, S., D. Nelson, M. Weiss, and M. Aepfelbacher. 1999. Wiskott-Aldrich syndrome protein regulates podosomes in primary human macrophages. *Proc. Natl. Acad. Sci. USA.* 96:9648–9653.

Lommel, S., S. Benesch, K. Rottner, T. Franz, J. Wehland, and R. Kühn. 2001. Actin pedestal formation by enteropathogenic *Escherichia coli* and intracellular motility of *Shigella flexneri* are abolished in N-WASP-defective cells. *EMBO Rep.* 2:850–857. http://dx.doi.org/10.1093/embo-reports/kve197

Misra, A., R.P.Z. Lim, Z. Wu, and T. Thanabalu. 2007. N-WASP plays a critical role in fibroblast adhesion and spreading. *Biochem. Biophys. Res. Commun.* 364:908–912. http://dx.doi.org/10.1016/j.bbrc.2007.10.086

Mizutani, K., H. Miki, H. He, H. Maruta, and T. Takenawa. 2002. Essential role of neural Wiskott-Aldrich syndrome protein in podosome formation and degradation of extracellular matrix in *src*-transformed fibroblasts. *Cancer Res.* 62:669–674.

Myers, S.A., J.W. Han, Y. Lee, R.A. Firtel, and C.Y. Chung. 2005. A *Dictyostelium* homologue of WASP is required for polarized F-actin assembly during chemotaxis. *Mol. Biol. Cell.* 16:2191–2206. http://dx.doi.org/10.1091/mbc.E04-09-0844

Sarmiento, C., W. Wang, A. Dovas, H. Yamaguchi, M. Sidani, M. El-Sibai, V. Desmarais, H.A. Holman, S. Kitchen, J.M. Backer, et al. 2008. WASP family members and formin proteins coordinate regulation of cell protrusions in carcinoma cells. *J. Cell Biol.* 180:1245–1260. http://dx.doi.org/10.1083/jcb.200708123

Shi, Y., B. Dong, H. Miliotis, J. Liu, A.S. Alberts, J. Zhang, and K.A. Siminovitch. 2009. Src kinase Hck association with the WASp and mDia1 cytoskeletal regulators promotes chemoattractant-induced Hck membrane targeting and activation in neutrophils. *Biochem. Cell Biol.* 87:207–216. http://dx.doi.org/10.1139/O08-130

Snapper, S.B., F. Takeshima, I. Antón, C.H. Liu, S.M. Thomas, D. Nguyen, D. Dudley, H. Fraser, D. Purich, M. Lopez-Ilasaca, et al. 2001. N-WASP deficiency reveals distinct pathways for cell surface projections and microbial actin-based motility. *Nat. Cell Biol.* 3:897–904. http://dx.doi.org/10.1038/ncb1001-897

Snapper, S.B., P. Meelu, D. Nguyen, B.M. Stockton, P. Bozza, F.W. Alt, F.S. Rosen, U.H. von Andrian, and C. Klein. 2005. WASP deficiency leads to global defects of directed leukocyte migration in vitro and in vivo. *J. Leukoc. Biol.* 77:993–998. http://dx.doi.org/10.1189/jlb.0804444

Tang, H., A. Li, J. Bi, D.M. Veltman, T. Zech, H.J. Spence, X. Yu, P. Timpson, R.H. Insall, M.C. Frame, and L.M. Machesky. 2013. Loss of Scar/WAVE complex promotes N-WASP- and FAK-dependent invasion. *Curr. Biol.* 23:107–117. http://dx.doi.org/10.1016/j.cub.2012.11.059

Veltman, D.M., J.S. King, L.M. Machesky, and R.H. Insall. 2012. SCAR knockouts in *Dictyostelium*: WASP assumes SCAR's position and upstream regulators in pseudopods. *J. Cell Biol.* 198:501–508. http://dx.doi.org/10.1083/jcb.201205058

Worth, A.J.J., J. Metelo, G. Bouma, D. Moulding, M. Fritzsche, B. Vernay, G. Charras, G.O.C. Cory, A.J. Thrasher, and S.O. Burns. 2013. Disease-associated missense mutations in the EVH1 domain disrupt intrinsic WASp function causing dysregulated actin dynamics and impaired dendritic cell migration. *Blood.* 121:72–84. http://dx.doi.org/10.1182/blood-2012-01-403857

Zhang, H., U.Y. Schaff, C.E. Green, H. Chen, M.R. Sarantos, Y. Hu, D. Wara, S.I. Simon, and C.A. Lowell. 2006. Impaired integrin-dependent function in Wiskott-Aldrich syndrome protein-deficient murine and human neutrophils. *Immunity.* 25:285–295. http://dx.doi.org/10.1016/j.immuni.2006.06.014

Zhu, Z., Y. Chai, Y. Jiang, W. Li, H. Hu, W. Li, J.-W. Wu, Z.-X. Wang, S. Huang, and G. Ou. 2016. Functional coordination of WAVE and WASP in *C. elegans* neuroblast migration. *Dev. Cell.* 39:224–238. http://dx.doi.org/10.1016/j.devcel.2016.09.029

Zicha, D., W.E. Allen, P.M. Brickell, C. Kinnon, G.A. Dunn, G.E. Jones, and A.J. Thrasher. 1998. Chemotaxis of macrophages is abolished in the Wiskott-Aldrich syndrome. *Br. J. Haematol.* 101:659–665.
